# A CD40-OX40 co-stimulatory circuit orchestrates protective CD4^+^ T cell immunity in tuberculosis

**DOI:** 10.1016/j.ymthe.2025.12.062

**Published:** 2026-01-03

**Authors:** Ximeng Zhang, Jing Yang, Fuxiang Li, Yu Wang, Zhaodong Li, Wenfei Wang, Yunlong Hu, Chenyan Shi, Fan Pan, Carl G Feng, Yejun Wang, Qianting Yang, Xinchun Chen, Yi Cai

**Affiliations:** 1 Guangdong Provincial Key Laboratory of Infection Immunity & Inflammation, Department of Pathogen Biology, Shenzhen University Medical School, Shenzhen 518000, China; 2 Laboratory Medicine, First Affiliated Hospital of Gannan Medical University, Ganzhou 341000, China; 3 National Clinical Research Center for Infectious Disease, Shenzhen Third People’s Hospital, Shenzhen 518000, China; 4 Faculty of Pharmaceutical Sciences, Shenzhen University of Advanced Technology, Shenzhen Institute of Advanced Technology, Chinese Academy of Sciences, Shenzhen 518000, China; 5 School of Medical Sciences, Faculty of Medicine and Health, The University of Sydney, Sydney, NSW 2006, Australia; 6 Department of Cell Biology and Genetics, Shenzhen University Medical School, Shenzhen 518000, China

**Keywords:** co-stimulatory signaling, OX40, CD40L, tuberculosis, host-directed therapy

## Abstract

Effective CD4^+^ T cell responses are essential for controlling *Mycobacterium tuberculosis* (*Mtb*), but they fail to achieve sterilizing immunity in infected lungs. In mouse models of *Mtb* infection, single-cell transcriptomic profiling identified a dominant population of OX40^+^ CD4^+^ T cells that emerged during *Mtb* infection yet exhibited impaired effector differentiation. This dysfunction was linked to defective co-stimulatory signaling, corresponding to persistently low OX40 ligand (OX40L) expression on antigen-presenting cells. Therapeutic activation of OX40 restored CD4^+^ T cell functionality, expanded protective clonotypes, and reduced pulmonary bacterial burden *in vivo*. These effects required intact CD40-CD40L interactions to sustain OX40 expression and promote T cell differentiation, and were critically dependent on interferon (IFN)-γ signaling for antimicrobial activity. CD40L blockade abolished the immunotherapeutic benefits of OX40 stimulation, revealing a cooperative CD40-OX40 axis that orchestrates protective immunity in tuberculosis. These findings identify impaired co-stimulatory signaling as a central mechanism of CD4^+^ T cell dysfunction during chronic infection and highlight this axis as a target for host-directed immunotherapy in tuberculosis.

## Graphical abstract

**Figure:**
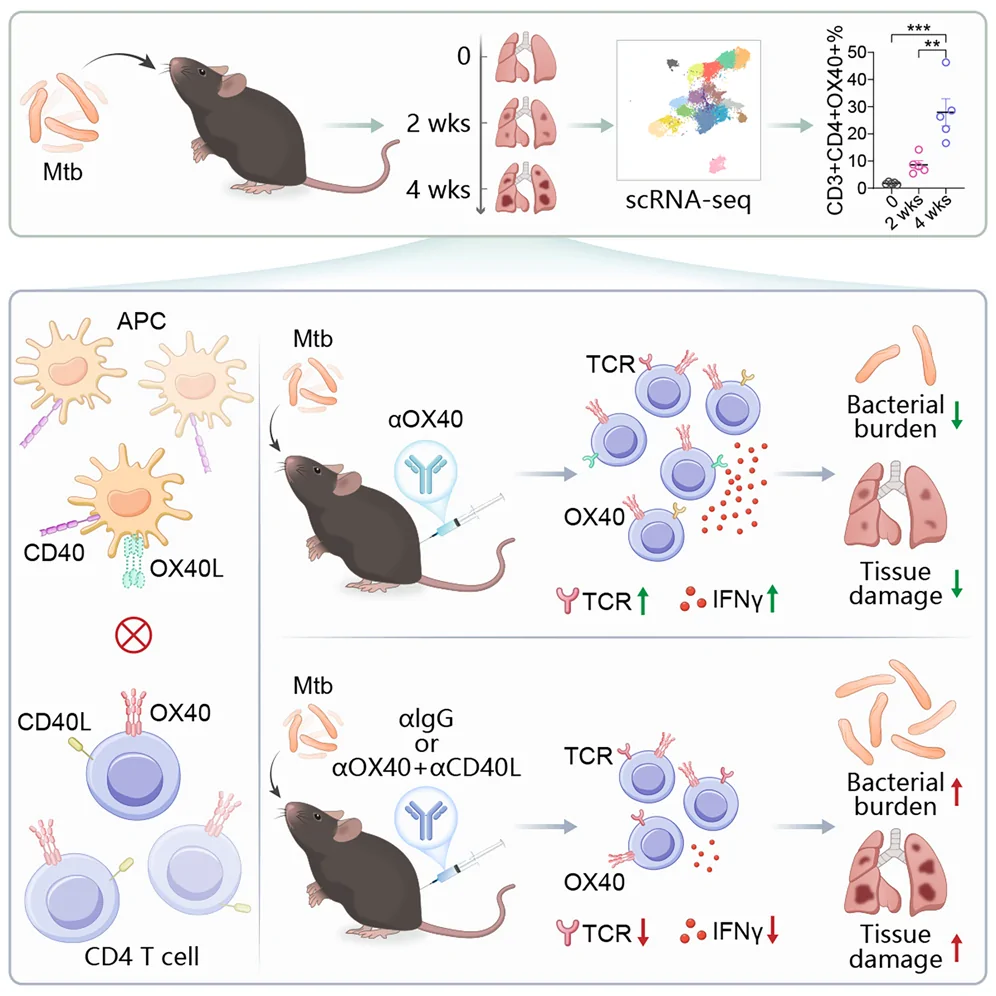

## Teaser

Single-cell analysis reveals that disrupted OX40-OX40L co-stimulation drives CD4^+^ T cell dysfunction during *Mycobacterium tuberculosis* infection. Restoring OX40 signaling enhances IFN-γ responses and bacterial clearance through CD40-dependent pathways, defining a CD40-OX40-IFN-γ axis for host-directed therapy in tuberculosis.

## Introduction

Tuberculosis (TB), caused by *Mycobacterium tuberculosis* (*Mtb*), is responsible for approximately 1.2 million deaths annually. In recent years, the emergence of drug-resistant *Mtb* strains and the high burden of human immunodeficiency virus (HIV) co-infection continue to undermine disease control efforts, highlighting the urgent need for more effective therapeutic strategies.^1^^,^^2^

A critical challenge in TB research lies in the incomplete understanding of how adaptive immune responses, particularly those mediated by CD4^+^ T cells, fail to achieve bacterial clearance in the lung.^3^^,^^4^ Protective immunity against *Mtb* requires the priming of naive CD4^+^ T cells through recognition of major histocompatibility complex (MHC) class II-restricted mycobacterial peptides presented by professional antigen-presenting cells (APCs), such as dendritic cells and macrophages. Upon T cell receptor (TCR) engagement and co-stimulatory signaling, these CD4^+^ T cells differentiate into T helper 1 (Th1) effector cells characterized by interferon (IFN)-γ production.^5^ IFN-γ is essential for activating macrophage antimicrobial programs, including phagolysosomal maturation, nitric oxide production, and autophagy, which are critical for intracellular control of *Mtb*.^6^ However, unlike many acute infections that elicit rapid and coordinated T cell responses,^7^^,^^8^ TB is marked by delayed T cell priming and frequent dysfunction.^4^^,^^9^^,^^10^ *Mtb* employs multiple immune-evasion strategies, including interference with MHC-II antigen presentation, modulation of dendritic cell migration and function, and inhibition of co-stimulatory molecule expression, which collectively impair efficient CD4^+^ T cell activation and expansion.^4^^,^^11^^,^^12^^,^^13^ Even when CD4^+^ T cells are recruited to the lungs, sustained antigenic stimulation in the context of chronic inflammation can drive functional exhaustion, metabolic dysregulation, and loss of effector capacity.^14^^,^^15^^,^^16^^,^^17^ Although CD4^+^ T cells are present in large numbers in the granulomatous lesions of infected lungs, their inability to clear infection suggests a qualitative defect in activation and maintenance. These limitations in T cell priming and sustained functionality represent key barriers to protective immunity and remain poorly defined at the mechanistic level.

Insights from cancer immunotherapy have highlighted the importance of immune checkpoint and co-stimulatory pathways in modulating T cell function.^18^^,^^19^ Several studies have reported that targeting these co-stimulatory pathways represents promising targets for host-directed therapy aimed at restoring T cell efficacy and improving outcomes in infectious diseases.^20^^,^^21^^,^^22^^,^^23^ In recent years, OX40 (TNFRSF4), a member of the tumor necrosis factor (TNF) receptor superfamily, has emerged as a critical regulator of T cell activation, survival, and effector differentiation.^24^ OX40 is predominantly expressed on activated CD4^+^ T cells following TCR engagement, while its ligand, OX40L, is primarily expressed on activated APCs.^25^ Engagement of the OX40-OX40L axis promotes T cell proliferation, cytokine production, and the generation of long-lived effector and memory populations.^24^ In *Mtb* infection, augmenting OX40 signaling enhances host resistance by promoting protective CD4^+^ T cell responses and reducing bacterial burden.^26^ The precise role of OX40 in TB immunity, however, remains incompletely understood. In particular, how OX40 signaling integrates with other immune-regulatory pathways, and whether T cell dysfunction in TB limits responsiveness to OX40-targeted interventions, remain open questions. A deeper understanding of OX40 dynamics in TB is critical for developing immunotherapeutic strategies to reverse immune dysfunction and to restore effective T cell-mediated protection.

Here, we aimed to determine how co-stimulatory signaling pathways contribute to CD4^+^ T cell dysfunction during TB, particularly during the early phase when adaptive immunity is initiated and CD4^+^ T cells begin to lose function, and whether restoring these signals could restore effector T cell functionality. Using single-cell transcriptomic and functional approaches, we focused on the OX40-OX40L axis, which has been implicated in protective CD4^+^ T cell responses. We further investigated whether its activity depends on upstream CD40-CD40L signaling and whether its modulation could enhance bacterial control. Our study uncovers a CD40-OX40 co-stimulatory circuit that sustains effector T cell function and reveals new opportunities for host-directed immunotherapy in TB.

## Results

### Single-cell profiling reveals dynamic immune cell remodeling and co-signaling deficits during *Mtb* infection

**Figure 1:**
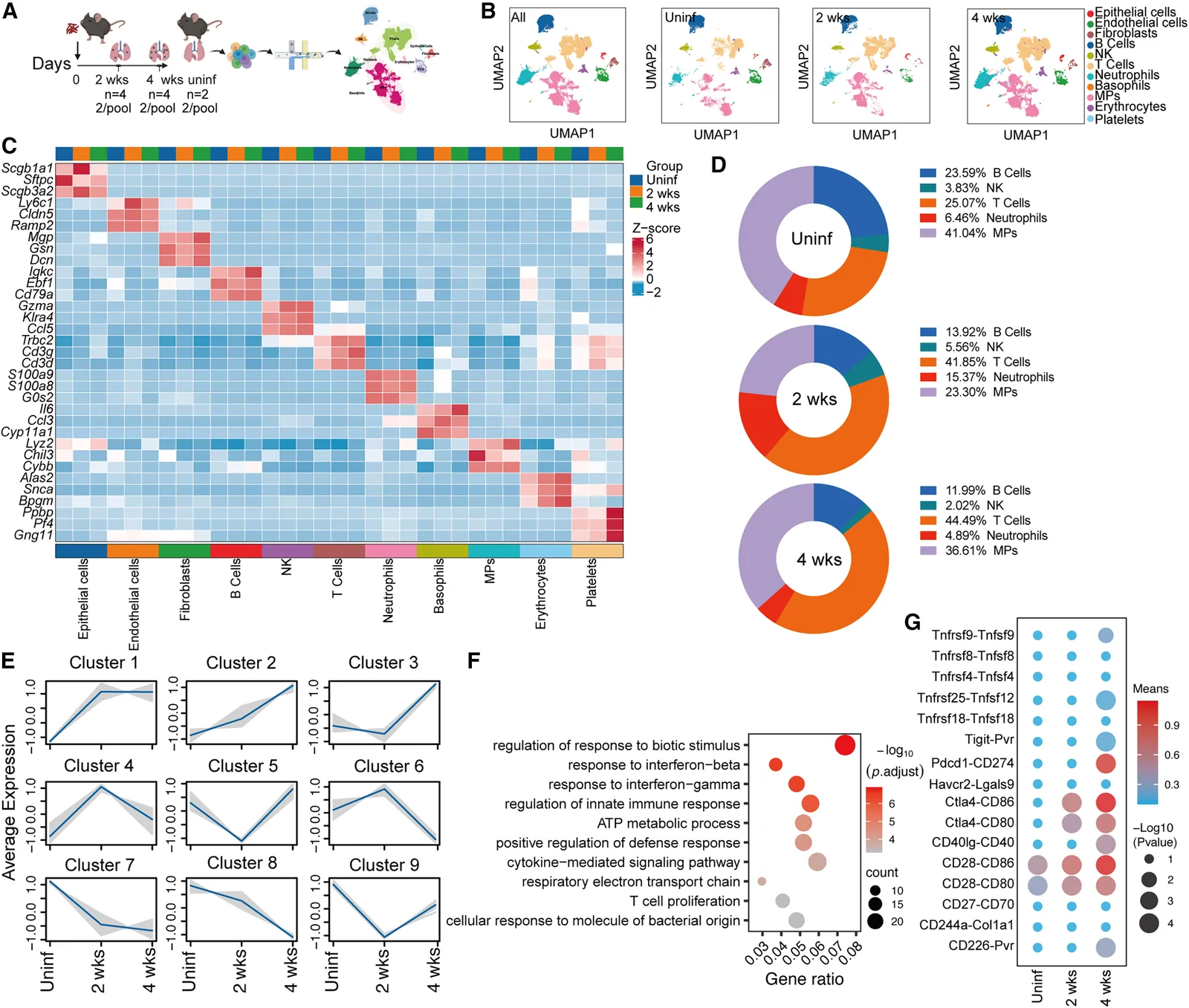
Single-cell transcriptomic profiling of lung immune landscapes during *Mtb* infection (A) Experimental workflow showing *Mtb* infection of C57BL/6 mice and lung tissue collection at uninfected, 2-week, and 4-week time points (*n* = 2 mice per pooled sample). (B) UMAP plots showing integrated clustering of lung immune and non-immune cells across all groups. (C) Heatmap displaying representative marker genes used for cell-type annotation across clusters. *Z* scores indicate normalized gene expression per cell type. (D) Donut plots showing proportional changes in major cell populations in uninfected, 2-week, and 4-week post-infection groups. (E) Temporal gene expression trajectory plots (nine clusters) for lung T cells the three conditions. (F) GO enrichment analysis of genes from cluster 1 in (E). Dot size indicates gene ratio; color scale reflects adjusted *p* values. (G) Ligand-receptor interaction analysis of T cells with myeloid populations showing differential expression of co-signaling pairs across infection stages. Gene symbols are shown according to official nomenclature. MPs, mononuclear phagocytes. *Ifng*, interferon-γ; *Pdcd1*, programmed cell death 1 (PD-1); *Cd40lg*, CD40 ligand. Receptor-ligand pairs in (G) include TNFRSF9 (CD137)-TNFSF9 (CD137L), TNFRSF4 (OX40)-TNFSF4 (OX40L), TNFRSF8 (CD30)-TNFSF8 (CD30L), TNFRSF18 (GITR)-TNFSF18 (GITRL), and TNFRSF25 (DR3)-TNFSF15 (TL1A).

To comprehensively characterize immune cell dynamics in the lungs during *Mtb* infection, we performed single-cell RNA sequencing (scRNA-seq) on lung-derived immune cells from uninfected mice and mice infected with *Mtb* at 2 or 4 weeks post infection. Single-cell suspensions were prepared from whole lung tissue, and CD45^+^ immune cells were enriched via magnetic bead separation before 10X Genomics-based scRNA-seq (Figure 1A). Unsupervised clustering identified 11 distinct cell populations based on canonical marker expression (Figures 1B and 1C; Table S1). Five clusters corresponded to non-immune cell types, including epithelial cells, endothelial cells, fibroblasts, erythrocytes, and platelets. Small erythrocyte and platelet clusters were detected, as reported in several lung single-cell datasets despite perfusion, and were excluded prior to downstream analyses. The remaining immune cell populations consisted of B cells, natural killer (NK) cells, neutrophils, basophils, mononuclear phagocytes (MPs), and T cells (Figure 1C). The composition of these immune cell populations changed dynamically over the course of *Mtb* infection. MPs decreased while neutrophils increased from week 2 to week 4, indicating innate remodeling alongside adaptive immune recruitment (Figure 1D). In particular, T cells increased markedly after infection (Figure 1D), reaching ∼40%–45% of lung immune cells at weeks 2–4. These findings confirm previous observations of adaptive immune recruitment during *Mtb* infection and provide single-cell resolution of stage-specific reshaping of the lung immune landscape.

We next examined how T cells transcriptionally responded across infection stages. Comparative analysis of T cells from uninfected, 2-week-, and 4-week-infected lungs revealed nine clusters of co-expressed genes with distinct temporal patterns (Figure 1E; Table S2). Among these, cluster 1 genes were upregulated during *Mtb* infection and enriched for pathways related to immune activation, pathogen sensing, and inflammatory signaling (Figure 1F). By contrast, cluster 7 genes were downregulated following infection and mapped to processes including T cell differentiation, metabolic regulation, and protein synthesis. Notable pathways included lymphocyte differentiation, αβ T cell differentiation, regulation of myeloid leukocyte differentiation, and cytoplasmic translation (Figure S1A). Downregulation of genes involved in response to glucose and carbohydrate stimuli suggests impaired metabolic adaptability. These findings point to a previously unreported paradoxical transcriptional state in T cells during *Mtb* infection. Effector genes are activated, yet key programs for differentiation (*Tcf7*, *Bach2*, *Foxp1*, *Zbtb20*) and metabolism (*Igf1r*, *Rras2*, *Adk*, *Amd1*) are suppressed, potentially reflecting an early onset of functional impairment.

Given the critical role of T cells in crosstalk with other immune cells through their co-signaling molecules,^27^ we next investigated whether disruptions in ligand-receptor interactions might underlie the impaired transcriptional state observed during *Mtb* infection. Ligand-receptor interaction analysis across time points revealed a marked disruption of co-stimulatory signaling, particularly among members of the TNF receptor (TNFR) superfamily (Figure 1G). In order to explore the co-signaling network, we examined the expression patterns of co-stimulatory molecules in both T cells and myeloid cells. Several key co-signaling ligand-receptor pairs, including *Tnfsf14/Tnfrsf14*, *Tnfrsf18/Tnfsf18*, and *Tnfsf9/Tnfrsf9*, were diminished or undetectable in both cell types during infection (Figures S1B and S1C). Notably, while T cells continued to express certain co-stimulatory receptors, including *Tnfrsf4* (encoding OX40), their corresponding ligands such as *Tnfsf4* (encoding OX40L) were markedly reduced in myeloid cells (Figure S1C). Among the co-stimulatory axes analyzed, the OX40-OX40L pair stood out due to its preserved receptor expression in T cells, its selective ligand loss in APCs, and its well-established relevance in promoting T cell survival and function. These features highlighted OX40 signaling as a candidate pathway warranting further mechanistic investigation.

### *Mtb* infection drives dynamic T cell differentiation and impairs co-stimulatory signaling in the lung

**Figure 2:**
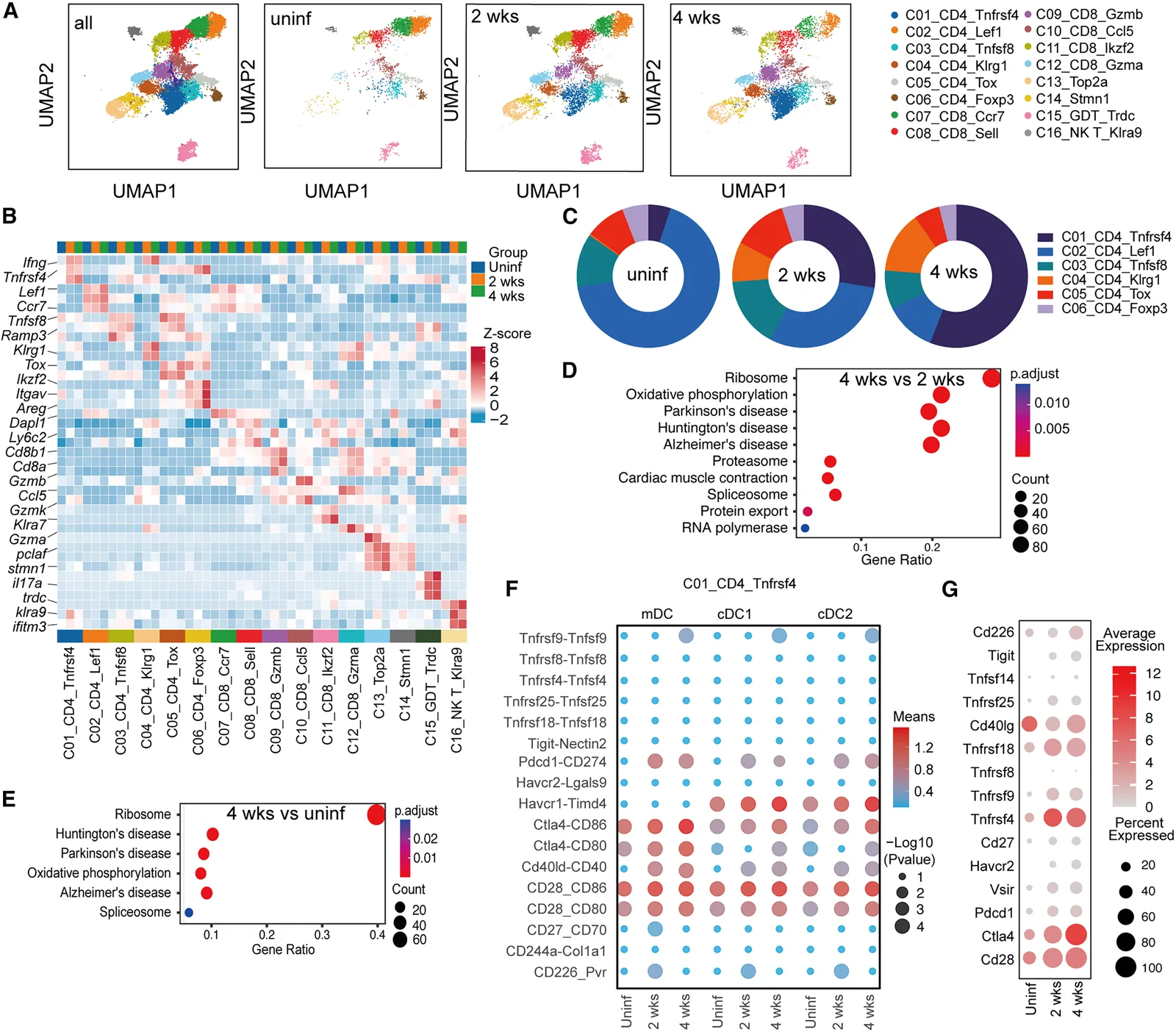
Single-cell analysis of T cell subsets in the lungs of *Mtb*-infected mice (A) UMAP projection of lung T cells from uninfected, 2-week, and 4-week *Mtb*-infected mice. (B) Heatmap of top marker genes for T/NK clusters at different time points, with the top two markers per cluster labeled. (C) Donut plots displaying the relative abundance of CD4^+^ T cell subsets (C01–C06) across different time points. (D) KEGG pathway-enrichment analysis of downregulated genes in C01_CD4_Tnfrsf4 cells at 4 weeks post infection compared to 2-week samples. (E) KEGG pathway-enrichment analysis of downregulated genes in C01_CD4_Tnfrsf4 cells at 4 weeks post infection compared to uninfected samples. (F) Ligand-receptor interaction analysis between C01_CD4_Tnfrsf4 and dendritic cell subsets (mDC, cDC1, cDC2). (G) Dot plot showing expression levels of selected co-signaling receptors in C01_CD4_Tnfrsf4 cells. Data are representative of pooled samples across replicates unless otherwise indicated. Gene abbreviations are defined in Figure 1. Additional genes shown here include *Lef1* (lymphoid enhancer-binding factor 1), *Klf9* (Kruppel-like factor 9), *Tox* (thymocyte selection-associated high-mobility group box protein), *Foxp3* (forkhead box P3), *Ikzf2* (Ikaros family zinc finger 2), *Gzma* (granzyme A), and *Gzmb* (granzyme B).

To further elucidate the intrinsic properties and functional potential of T cell populations during *Mtb* infection, we performed unsupervised clustering of lung-derived T cells, identifying six distinct CD4^+^ clusters, six CD8^+^ clusters, and two unconventional T cell clusters (Figure 2A; Table S3). These subclusters were defined based on their transcriptional signatures (Figure 2B). The composition and relative proportions of these subsets changed dynamically over the course of infection. Naive T cell subsets (C02, C07, and C08) progressively declined, indicating a transition toward activated and memory phenotypes. By contrast, cytotoxic Klrg1^+^ CD4^+^ T cells (C04) and CD8^+^ T cells (C09) were enriched at 4 weeks post infection, suggesting expansion of effector responses over time. CD4^+^ Tox^+^ (C05) and IKZF2^+^ CD8^+^ (C11) T cells were predominantly detected at 2 weeks, implying a role in early-stage responses. Two unconventional T cell clusters (C13 and C14) also expanded significantly following infection. Notably, one CD4^+^ T cell cluster (C01) emerged as the dominant subset within the CD4^+^ compartment by 4 weeks post infection, comprising the largest proportion of CD4^+^ T cells among all identified clusters (Figure 2C; Figure S2A). This subset exhibited elevated expression of multiple genes associated with T cell activation and effector function. Among these, *Tnfrsf4* and *Ifng* were prominently upregulated, both of which are known to play critical roles in CD4^+^ T cell co-stimulation and antimicrobial activity, respectively (Figure 2B). These genes were highlighted based on their established immunological relevance and their rank among the top differentially expressed markers within this cluster.

To gain further insight into the differentiation trajectories of CD4^+^ and CD8^+^ T cell subsets, we conducted pseudotime analysis. CD4^+^ T cell differentiation appeared to originate from the naive-like C02 cluster and diverged along three major branches (Figure S2B). C05_CD4_Tox emerged as a transient intermediate state that subsequently gave rise to C04, C06, and C01. Although TOX expression is commonly associated with dysfunction, analysis of C05 showed that inhibitory receptor co-expression (*Pdcd1*, *Havcr2*, and *Lag3*) was infrequent (Figure S2C), supporting that this cluster represents a pre-exhausted or transitional state rather than terminal exhaustion. CD8^+^ T cell differentiation was initiated from the naive-like C07 cluster and progressed along two distinct trajectories, ultimately giving rise to differentiated effector subsets (Figure S2D).

Given that C01_CD4_Tnfrsf4 represented the dominant CD4^+^ T cell subset during *Mtb* infection and displayed a terminally differentiated phenotype, we next characterized its functional state. Gene Ontology (GO) analysis revealed a transcriptional program indicative of emerging dysfunction, with upregulated genes enriched in TCR signaling and pathogen response pathways, whereas downregulated genes were associated with T cell activation and differentiation (Figures S2E and S2F). Kyoto Encyclopedia of Genes and Genomes (KEGG) pathway analysis further highlighted suppression of metabolic programs, including fatty acid, linoleic acid, and arachidonic acid metabolism (Figure S2G).

To investigate the temporal evolution of this phenotype, we examined transcriptional changes in C01_CD4_Tnfrsf4 cells across different infection stages. By 4 weeks post infection, this subset showed progressive downregulation of oxidative phosphorylation and ribosomal biogenesis relative to both 2-week and uninfected controls (Figures 2D and 2E), suggesting that chronic antigenic stimulation imposes cumulative bioenergetic and translational constraints. Together, these results indicate that C01_CD4_Tnfrsf4 cells undergo metabolic alterations and may be entering early stages of functional impairment during *Mtb* infection.

Building on the global T cell alterations described in Figure 1, we next sought to identify which CD4^+^ T cell subsets were most affected by co-signaling disruption. Ligand-receptor interaction analysis specifically within the dominant C01_CD4_Tnfrsf4 cluster revealed attenuated engagement with myeloid populations, including macrophages, monocytes, and dendritic cells (Figure 2F; Figures S2H–S2J). While most TNFR superfamily receptors and their ligands were expressed at low levels across both T cells and APCs (e.g., Tnfrsf18/Tnfsf18, Tnfrsf25/Tnfsf15), OX40 was highly expressed in C01_CD4_Tnfrsf4 cells (Figure 2G). However, its ligand OX40L remained at very low and infrequent levels across APC subsets and similarly low expression was observed in non-APC immune populations (Figures S2K–S2L).

This cell-type-specific asymmetry, now resolved at the subset level, reinforces the hypothesis that OX40-mediated co-stimulation is selectively impaired in the key CD4^+^ T cell population that expands during *Mtb* infection, potentially undermining effective T cell priming and effector differentiation.

### Restoring OX40 signaling reverses T cell dysfunction and enhances immunity in tuberculosis

**Figure 3:**
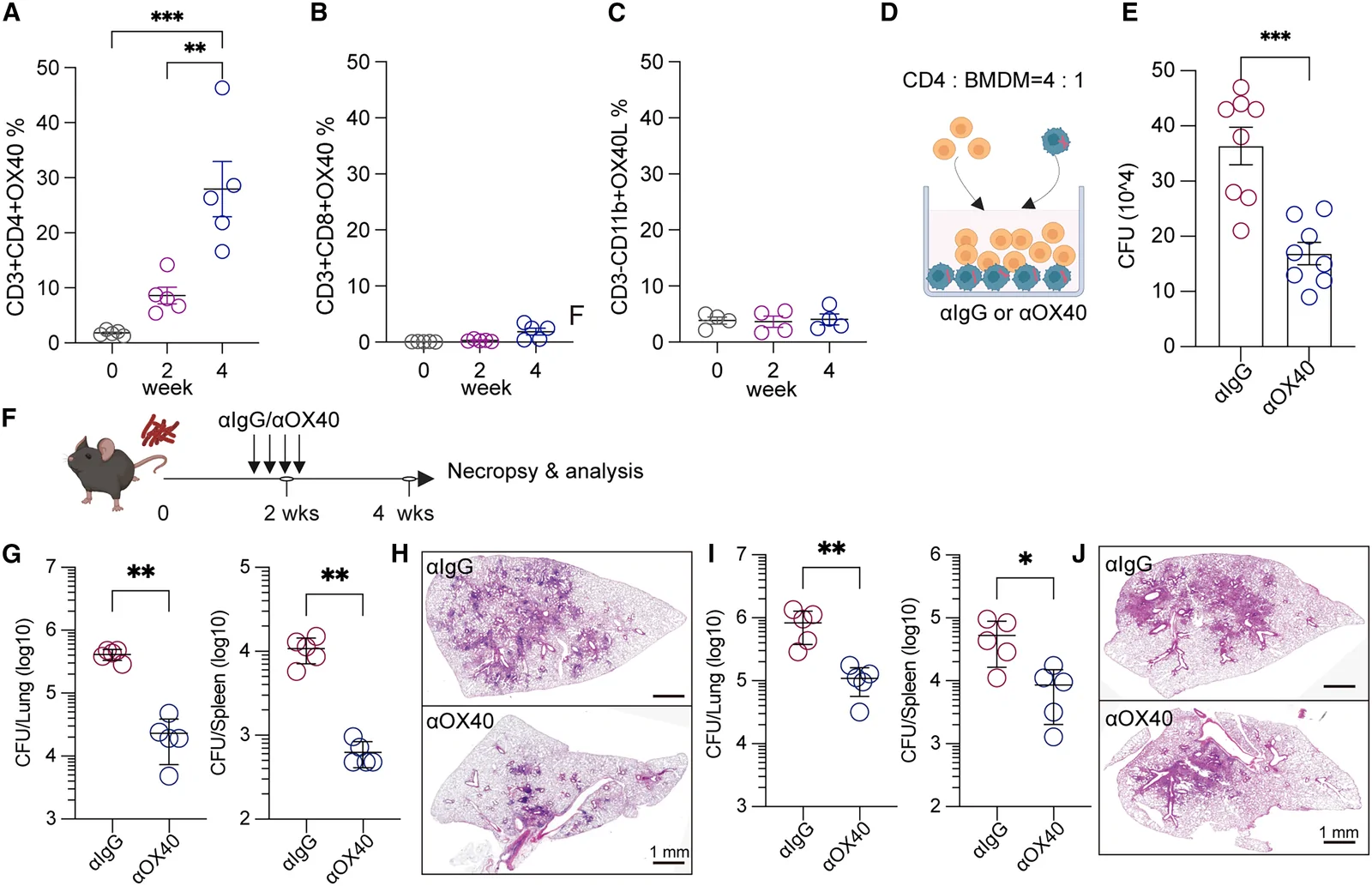
OX40 agonist treatment promotes CD4^+^ T cell activation and enhances bacterial control in *Mtb*-infected mice (A–C) Flow cytometric analysis of OX40 and OX40L expression in lung immune subsets from C57BL/6 at 0, 2, and 4 weeks post infection (*n* = 4). (A) OX40 expression in CD4^+^ T cells. (B) OX40 expression in CD8^+^ T cells. (C) OX40L expression in CD11b^+^ myeloid cells. (D and E) *In vitro* co-culture assay schematic (D) and quantification of intracellular *Mtb* burden (E) in infected BMDMs co-cultured with lung-derived CD4^+^ T cells from C57BL/6 in the presence or absence of αOX40 stimulation (*n* = 8). (F) Schematic of *in vivo* αOX40 treatment regimen in C57BL/6 and C3H mice. Antibody was administered on days 10, 12, 14, and 16 post infection, with analysis at 4 weeks. (G) Bacterial loads in the lungs and spleens from C57BL/6 mice treated with αOX40 or αIgG (*n* = 5). (H) Representative H&E-stained lung sections from C57BL/6 mice treated with αOX40 or αIgG. (I) Bacterial loads in the lungs and spleens from C3H mice treated with αOX40 or αIgG (*n* = 5). (J) Representative H&E-stained lung sections from C3H mice treated with αOX40 or αIgG. Data are presented as mean ± SEM. Statistical significance was assessed using unpaired *t* test for two-group comparisons and one-way ANOVA for comparisons among three or more groups. ∗*p* < 0.05, ∗∗*p* < 0.01, ∗∗∗*p* < 0.001. αOX40, anti-OX40 agonistic antibody; αIgG, isotype control antibody; CFU, colony-forming units.

Building on the finding that expansion of OX40^+^ CD4^+^ T cells during *Mtb* infection, we next sought to validate the expression dynamics of key upregulated co-signaling molecules at the protein level. Flow cytometric analysis revealed sustained upregulation of OX40 in CD4^+^ T cells, with elevated expression persisting up to 4 weeks post infection (Figure 3A; Figure S3A). By contrast, OX40 expression remained undetectable in CD8^+^ T cells, and its ligand, OX40L, was largely absent from CD11b^+^ myeloid cells, suggesting a specific disruption of the OX40-OX40L axis during infection (Figures 3B and 3C; Figure S3B).

To assess the functional consequences of impaired co-signaling interactions, we examined the responsiveness of OX40^+^ CD4^+^ T cells to OX40-mediated stimulation. OX40^+^ CD4^+^ T cells were isolated from the lungs of *Mtb*-infected mice at 4 weeks post infection and co-cultured with *Mtb*-infected bone marrow-derived macrophages (BMDMs) in the presence of either an OX40 agonist (αOX40) or an αIgG control (Figure 3D). OX40 stimulation significantly enhanced bacterial clearance relative to control conditions (Figure 3E), suggesting that OX40 signaling augments the antimicrobial function of CD4^+^ T cells.

Because OX40 expression on CD4^+^ T cells increased markedly by 2 weeks post infection, αOX40 treatment was initiated on day 10 to coincide with the emergence and expansion of OX40^+^ T cells. To evaluate its therapeutic potential *in vivo*, both C57BL/6 and C3H mouse models were used (Figure 3F). C57BL/6 mice, which are relatively resistant to *Mtb* infection and typically exhibit lower bacterial burdens and milder lung pathology, were treated with αOX40 on days 10, 12, 14, and 16 post infection. This treatment regimen resulted in a marked reduction in bacterial loads in both the lungs and spleens compared to αIgG-treated controls (Figure 3G). Histological analysis further revealed reduced pulmonary inflammation in αOX40-treated animals (Figure 3H), supporting the protective effect of OX40 stimulation in this model. To validate these findings in a more susceptible host, we performed the same αOX40 treatment schedule in C3H mice, which develop more severe disease upon *Mtb* infection.^28^ αOX40-treated C3H mice similarly demonstrated significantly lower bacterial burdens and reduced pulmonary pathology (Figures 3I and 3J), highlighting the robust protective effects of OX40 activation across distinct genetic backgrounds.

Given that OX40L expression was very low and infrequent during *Mtb* infection *in vivo*, we next investigated whether this deficiency directly contributes to impaired OX40-mediated signaling. To test this, *Mtb*-infected mice were treated with αOX40, an OX40L-blocking antibody (αOX40L), or αIgG control (Figure S4A). As expected, αOX40 treatment significantly reduced bacterial burden in both the lungs and spleens relative to controls, whereas αOX40L blockade had no measurable effect compared to αIgG-treated mice (Figures S4B and S4C). Consistent with these findings, histopathological analysis showed that αOX40-treated animals displayed markedly reduced pulmonary inflammation and tissue damage, while αOX40L blockade did not improve pathology and even led to slightly greater inflammation than IgG controls, possibly reflecting the limited contribution of residual low-level OX40L expression (Figures S4D and S2K). Together, these results support that impaired OX40 signaling during *Mtb* infection is primarily due to insufficient OX40L expression, rather than intrinsic defects within OX40^+^ CD4^+^ T cells. The absence of functional ligand engagement likely represents a critical barrier limiting OX40-dependent immune activation and bacterial clearance *in vivo*.

### OX40 stimulation drives IFN-γ^+^ CD4^+^ T cell responses required for host protection against TB

**Figure 4:**
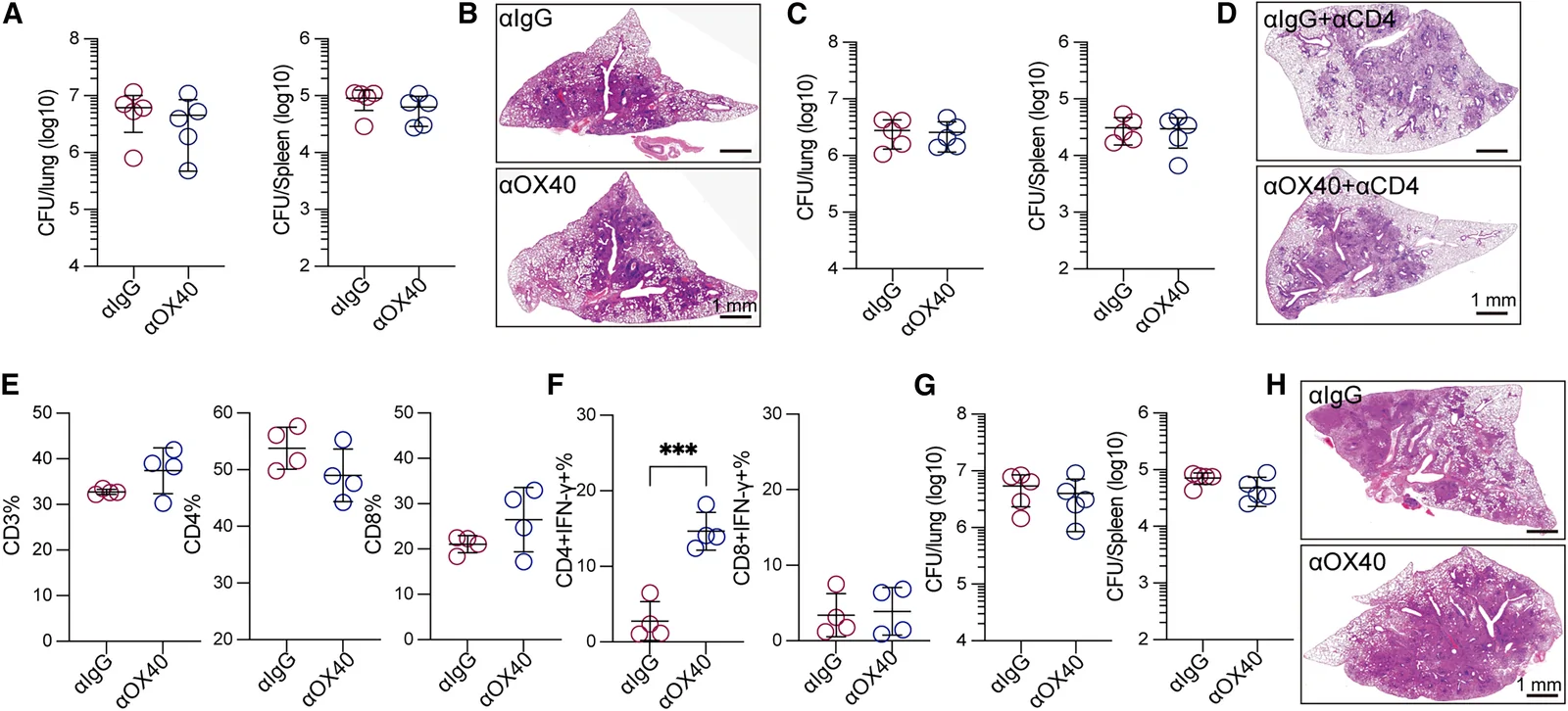
Analysis of OX40 agonist treatment in various immunodeficient mouse models during *Mtb* infection (A) Bacterial loads in the lungs and spleens from *Rag1*^−/−^ mice treated with αOX40 or αIgG (*n* = 5). (B) Representative H&E-stained lung sections from *Rag1*^−/−^ mice treated with αOX40 or αIgG. (C) Bacterial loads in the lungs and spleens from CD4-depleted C57BL/6 mice treated with αOX40 or αIgG (*n* = 5). (D) Representative H&E-stained lung sections from CD4-depleted C57BL/6 mice treated with αOX40 or αIgG. (E) Frequencies of lung CD3^+^, CD4^+^, and CD8^+^ T cells in *Mtb*-infected C57BL/6 mice treated with αOX40 or αIgG (*n* = 4). (F) Frequency of lung IFN-γ-producing CD4^+^ and CD8^+^ T cells in *Mtb*-infected C57BL/6 mice treated with αOX40 or αIgG (*n* = 4). (G) Bacterial loads in the lungs and spleens from *Ifngr*^−/−^ mice treated with αOX40 or αIgG (*n* = 5). (H) Representative H&E-stained lung sections from *Ifngr*^−/−^ mice treated with αOX40 or αIgG. Data are presented as mean ± SEM. Statistical analysis was performed using unpaired *t* test. ∗∗∗*p* < 0.001. αCD4, anti-CD4-depleting antibody.

To determine whether adaptive immunity is required for the protective effects of OX40 activation during *Mtb* infection, we used Rag1-knockout (*Rag1*^−/−^) mice,^29^ which allows us to test whether αOX40 acts through the adaptive immune system, as any protection observed in *Rag1*^−/−^ mice would necessarily result from innate immunity or off-target mechanisms. Consistent with the treatment timeline in immunocompetent models, αOX40 was administered on day 10 post infection, and outcomes were assessed at 4 weeks. αOX40 treatment failed to reduce bacterial burden or improve lung pathology in *Rag1*^−/−^ mice compared to αIgG-treated controls (Figures 4A–4C), indicating that OX40-mediated protection critically depends on adaptive immune components.

Given that OX40 is predominantly expressed on CD4^+^ T cells, we next tested whether these cells mediate the protective effects of αOX40. CD4^+^ T cells were depleted in *Mtb*-infected mice prior to αOX40 administration, and flow cytometry confirmed efficient depletion following antibody treatment (Figures S5A–S5C). CD4^+^ T cell depletion completely abolished the therapeutic benefit of αOX40, with bacterial burdens that were indistinguishable between αOX40- and αIgG-treated groups (Figures 4D–4F). These results identify CD4^+^ T cells as the primary effector population through which OX40 signaling confers protection during TB.

We then asked whether αOX40 treatment selectively expands functional CD4^+^ T cell subsets. Flow cytometric analysis revealed no significant changes in the overall frequencies of CD3^+^, CD4^+^, or CD8^+^ T cells, or in myeloid populations, following αOX40 treatment (Figure 4G; Figures S5D and S5E); however, αOX40 significantly increased the frequency of IFN-γ-producing CD4^+^ T cells, while IFN-γ expression in CD8^+^ T cells remained unchanged (Figure 4H). To confirm the functional relevance of this response, we administered αOX40 to IFN-γ receptor knockout (*Ifngr1*^−/−^) mice. In the absence of IFN-γ signaling, αOX40 failed to reduce bacterial burden, with outcomes comparable to αIgG-treated controls (Figures 4I–4K). Together, these findings demonstrate that OX40 stimulation improves host resistance to *Mtb* by expanding and activating IFN-γ-producing CD4^+^ T cells. This protective effect requires intact IFN-γ signaling.

### CD40-CD40L signaling sustains OX40-driven protective CD4^+^ T cell responses during *Mtb* infection

**Figure 5:**
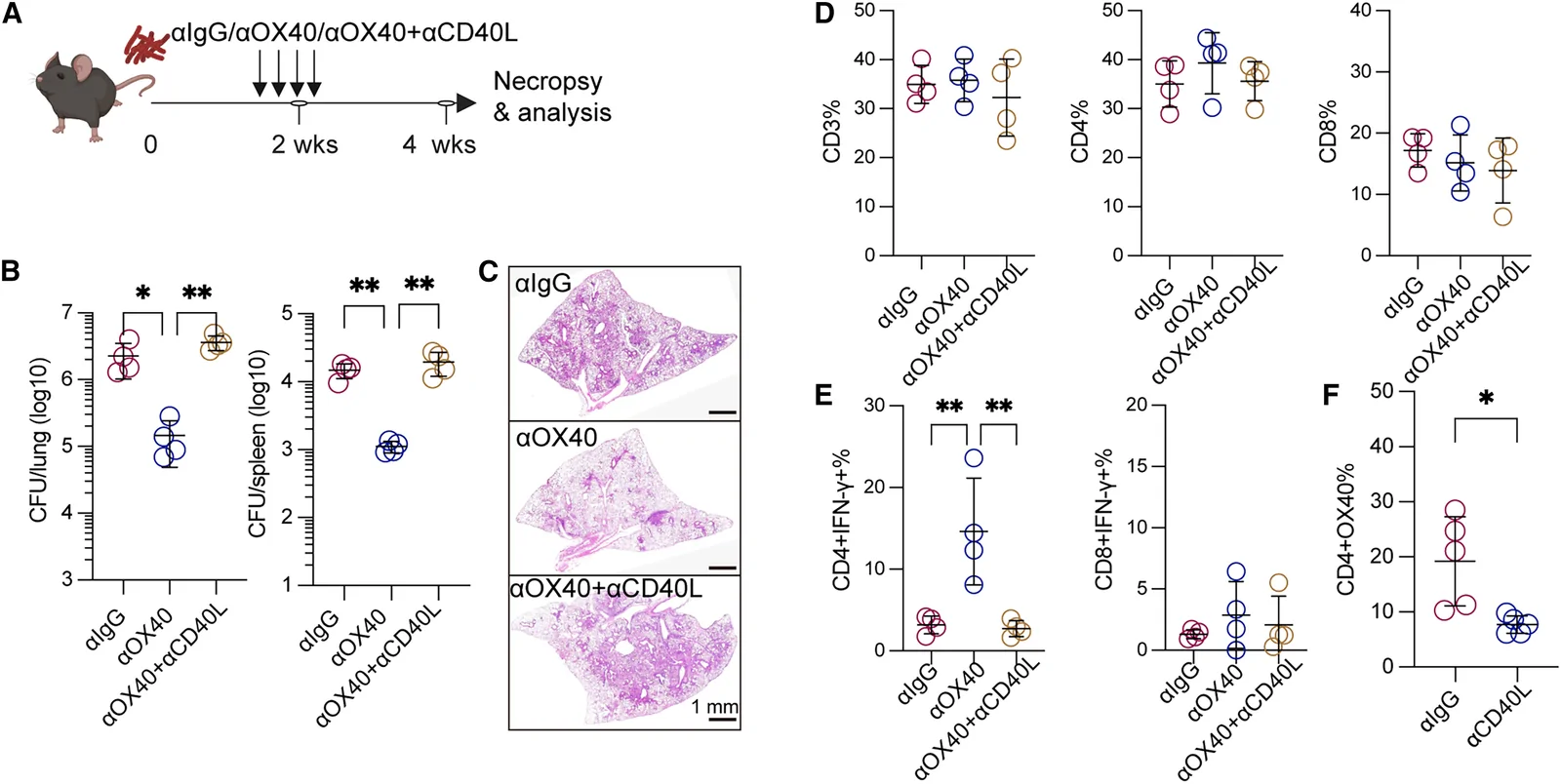
Assessment of immune responses and bacterial burden following αOX40 and αCD40L treatment during chronic *Mtb* infection (A) Experimental timeline showing treatment with αIgG, αOX40, or combined αOX40 and αCD40L during *Mtb* infection. (B) Lung and spleen CFU count across groups (*n* = 4). (C) Representative H&E-stained lung sections showing histopathological changes in each group. (D) Frequencies of CD3^+^, CD4^+^, and CD8^+^ T cells among lung leukocytes (*n* = 4). (E) Frequencies of IFN-γ-producing CD4^+^ and CD8^+^ T cells in lung single-cell suspensions following *ex vivo* restimulation (*n* = 4). (F) Proportion of OX40^+^ cells among lung CD4^+^ T cells at 4 weeks post infection in mice treated with αIgG or αCD40L (*n* = 4). Data are shown as mean ± SEM. Statistical significance was assessed using unpaired *t* test for two-group comparisons and one-way ANOVA for comparisons among three or more groups. ∗*p* < 0.05, ∗∗*p* < 0.01. αCD40L, anti-CD40L blocking antibody.

OX40 expression on T cells is primarily induced by TCR stimulation and further amplified by cytokines and co-stimulatory signals, including the CD28-B7 and CD40L-CD40 pathways.^30^ Because OX40 activation enhances IFN-γ^+^ CD4^+^ T cell responses and the observed co-expression of OX40 and CD40L on C01_CD4_Tnfrsf4 cells (Figure 2G), we tested whether CD40-CD40L signaling is required for this activity. We administered a CD40L-blocking antibody (αCD40L) in combination with αOX40 to *Mtb*-infected mice (Figure 5A). CD40L blockade abolished the protective effect of OX40 stimulation, resulting in increased bacterial burdens in the lungs and spleens and more severe pathology compared to αOX40 treatment alone (Figures 5B and 5C). For clarity, αCD40L monotherapy data are shown in the supplemental information (Figures S6A and S6B). Consistent with a previous study, αCD40L alone had no detectable effect on bacterial burden.^31^

Interestingly, the loss of OX40-mediated protection in αOX40 + αCD40L-treated mice was not associated with changes in total T cell frequencies (Figure 5D). Rather, IFN-γ production was selectively and significantly reduced in CD4^+^ T cells, while CD8^+^ T cell IFN-γ expression remained unchanged (Figure 5E). These findings indicate that CD40-CD40L signaling specifically sustains OX40-driven IFN-γ responses in CD4^+^ T cells, revealing a selective and previously unappreciated interdependence, on this pathway for CD4^+^, but not CD8^+^, T cell cytokine production during *Mtb* infection.

To determine whether CD40-CD40L signaling influences not only OX40-mediated function but also the expression of OX40 itself, we examined OX40 surface levels on lung-derived CD4^+^ T cells following αCD40L treatment. This experiment aimed to assess whether upstream co-stimulation through the CD40 pathway is required to sustain OX40 surface expression during infection. Flow cytometric analysis revealed a significant reduction in OX40 expression in αCD40L-treated mice compared to αIgG controls (Figure 5F). These results demonstrate that intact CD40-CD40L signaling is essential for functional T cell activation and maintaining OX40 receptor expression, reinforcing its role as a prerequisite for effective OX40-driven immunity during *Mtb* infection.

### OX40 drives CD4^+^ T cell clonal expansion and TCR diversity via CD40/CD40L-dependent programming

**Figure 6:**
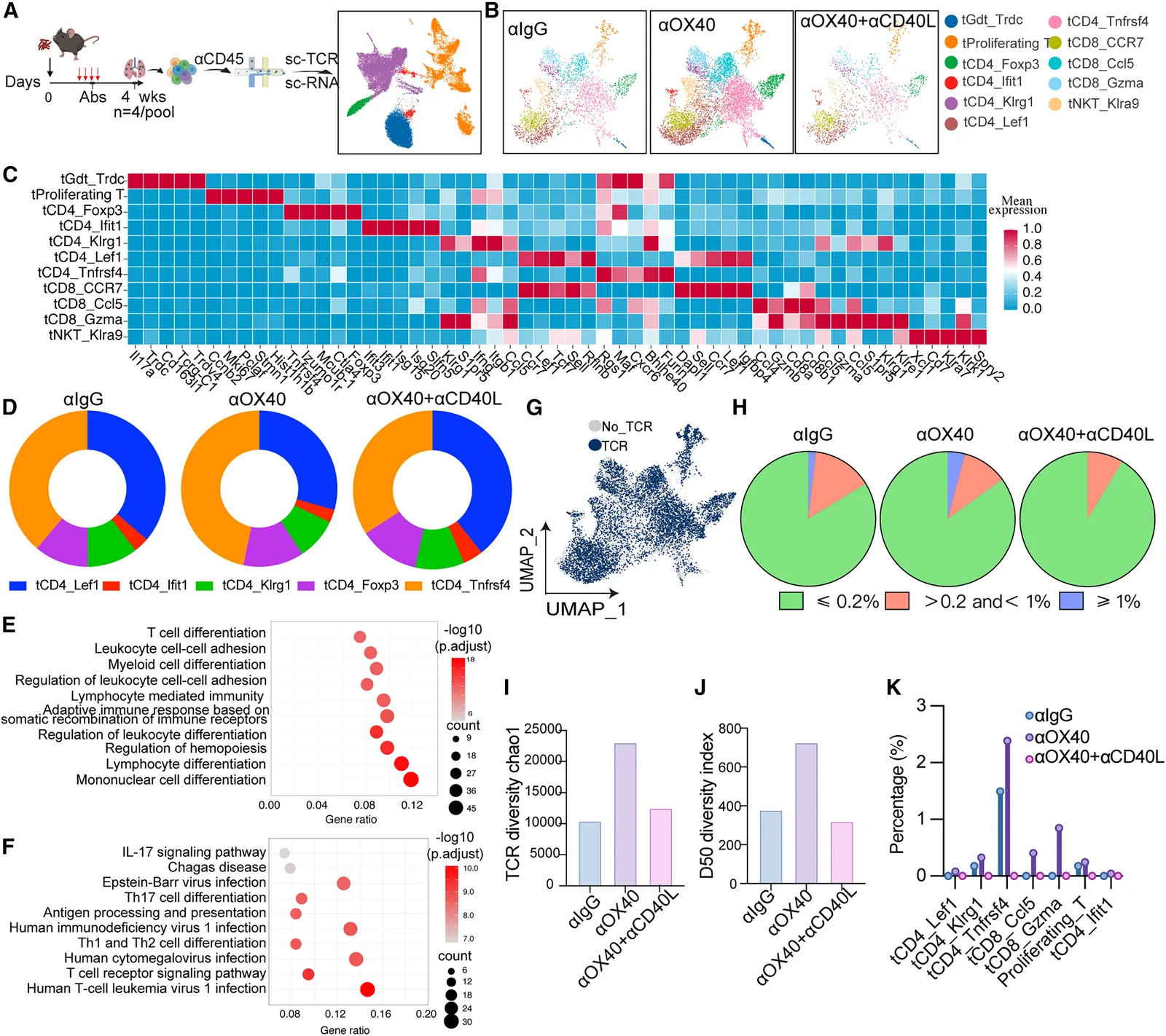
Single-cell transcriptomic and TCR repertoire analysis of lung T cells following OX40 stimulation and antagonistic CD40L treatment during *Mtb* infection (A) Experimental timeline for antibody treatment and lung cell preparation for scRNA-seq and scTCR-seq (pooled samples, *n* = 4). (B) UMAP plots showing clustering of lung T cells from *Mtb*-infected mice treated with αIgG, αOX40, or αOX40 + αCD40L, colored by annotated T cell subsets. (C) Heatmap displaying mean expression levels of representative genes across identified T cell clusters. (D) Donut plots showing the proportional distribution of CD4^+^ T cell subsets across treatment groups. (E) GO enrichment analysis of genes upregulated in the tCD4_Tnfrsf4 subset from αOX40-treated versus αOX40 + αCD40L-treated mice. (F) KEGG pathway-enrichment analysis of the same upregulated gene set. (G) UMAP plot showing TCR detection status at the single-cell level. (H) Clonotype distribution across treatment groups categorized by relative clone size. Clonotypes were grouped based on their proportional abundance within each sample: small (<0.2%), medium (0.2%–1%), and large (≥1%) of total TCR-expressing cells. (I and J) TCR repertoire diversity quantified by Chao1 index (I) and D50 diversity index (J). (K) Frequency of large TCR clones (≥1%) across T cell clusters in each treatment group. Data were obtained from scRNA-seq and paired scTCR-seq analysis of lung CD3^+^ T cells at 4 weeks post infection. The “t” prefix marks clusters from the treatment dataset. αCD45, anti-CD45 antibody; scRNA, single-cell RNA; scTCR, single-cell T cell receptor; TCR, T cell receptor.

To further elucidate how OX40 signaling influences T cell responses during *Mtb* infection, we performed scRNA-seq and single-cell TCR sequencing (scTCR-seq) of lung-derived T cells from infected mice treated under different conditions at 4 weeks post infection (Figure 6A). Based on marker genes, we identified several cell-type clusters that could be defined to specific immune cell populations (Figures S7A–S7C; Table S4).

To examine treatment-specific effects within the T cell compartment, we performed unsupervised clustering and identified 11 transcriptionally distinct T cell subclusters (Figures 6B and 6C; Table S5). Notably, the αOX40-treated group exhibited selective expansion of the CD4^+^ OX40^+^ (tCD4_Tnfrsf4) subset (Figure 6D; Figure S7D). To characterize transcriptional changes in this subset, we performed GO enrichment analyses on upregulated genes following αOX40 treatment. Compared to αIgG controls, αOX40 induced strong enrichment in pathways related to T cell differentiation, somatic recombination, immune adhesion, and homeostatic regulation, indicating enhanced adaptive immune programming and tissue retention (Figure S7E). KEGG analysis further revealed enhanced signaling in TCR, TNF, and Th1/Th17 pathways, consistent with increased effector function and inflammatory potential (Figure S7F).

We next examined the impact of CD40L blockade on OX40-mediated transcriptional reprogramming. Compared to the αOX40 group, the αOX40 + αCD40L-treated group showed a marked reduction in the expression of genes involved in lymphocyte activation, leukocyte adhesion, and positive regulation of immune response (Figure 6E), indicating that CD40/CD40L signaling is required to sustain the immunomodulatory effects of OX40 stimulation. Consistently, KEGG analysis demonstrated a loss of OX40-induced enrichment in pathways such as IL-17 signaling, Th1/Th17 differentiation, antigen processing and presentation, and TCR signaling in the presence of CD40L blockade (Figure 6F). These findings suggest that OX40 stimulation promotes adaptive immune activation at the transcriptional level. However, its efficacy depends on intact CD40/CD40L signaling, which appears necessary to reinforce or stabilize key effector programs downstream of OX40 engagement.

To assess the effect of OX40 signaling on TCR repertoire dynamics, we analyzed scTCR-seq data from αIgG, αOX40, and αOX40 + αCD40L treatment groups, recovering 4,330 paired full-length TCR α- and β-chain sequences (Figure 6G; Table S6). Clonal expansion, defined as clonotypes containing >2 cells and representing ≥0.2% of all TCR clonotypes within each sample, was observed across all groups. However, large clonal expansions (≥1% of TCR clonotypes) were predominantly observed in the αOX40 group, accounting for 4.1% of all expanded clonotypes, compared to 1.8% in the αIgG group and none in the αOX40 + αCD40L group (Figure 6H). TCR diversity, measured by the Chao1 richness and the D50 index, was highest in αOX40-treated mice compared to both controls and CD40L-blocked mice (Figures 6I and 6J). Supporting these findings, GLIPH2 analysis^32^ identified 144 clonotype groups in αOX40-treated mice, versus only 40 and 11 groups in αIgG and αOX40 + αCD40L cohorts, respectively (Figure S7G; Table S7).

Notably, the largest clonal expansions arose predominantly from the tCD4_Tnfrsf4 subset after αOX40 treatment (Figure 6K), indicating that αOX40 treatment selectively drives the expansion of antigen-responsive OX40^+^ CD4^+^ T cells. A smaller proportion of expanded clonotypes was detected in the tCD8_GZMA subset, likely reflecting secondary expansion via CD4^+^ T cell-derived helper signals rather than direct OX40 engagement. Minimal TCR overlap between tCD4_Lef1 and tCD4_Tnfrsf4 subsets in the αOX40 group (Figure S7H) further supports the conclusion that OX40 primarily drives local expansion of antigen-experienced T cells rather than *de novo* differentiation from naive precursors.

Together, these findings show that OX40 signaling enhances TCR repertoire diversity and promotes robust, clonotype-specific expansion predominantly within the tCD4_Tnfrsf4 compartment and that these effects critically depend on intact CD40/CD40L co-stimulatory interactions.

## Discussion

Despite sustained antigen exposure during TB, CD4^+^ T cells often fail to mount effective protective responses, a central paradox that remains poorly understood. This study focused on the early to mid phase of infection, when CD4^+^ T cell dysfunction first emerges, using single-cell transcriptomics to define the mechanisms of impaired co-stimulation. Although CD4^+^ T cells progressively accumulated in *Mtb*-infected lungs, they exhibited diminished effector function, which correlated with very low OX40L expression on myeloid APCs. This impaired receptor-ligand interaction appears to undermine CD4^+^ T cell activation despite antigen recognition. Moreover, we show that OX40-mediated protection depends critically on intact CD40/CD40L signaling, revealing key crosstalk between these co-stimulatory pathways. Together, these findings identify defective co-stimulation as a major contributor to T cell dysfunction in TB and support the therapeutic potential of targeting the OX40-CD40L axis to restore protective immunity.

Previous studies have shown that CD4^+^ T cell dysfunction facilitates bacterial persistence and reactivation during *Mtb* infection.^14^^,^^33^^,^^34^ This impairment may form a self-reinforcing loop, wherein suboptimal immunity promotes continued replication and disease progression.^14^ Our findings advance this understanding by identifying the OX40-OX40L axis as a critical co-stimulatory checkpoint that is selectively disrupted in the *Mtb*-infected lung, thereby impairing CD4^+^ T cell activation despite sustained antigen exposure. Although OX40^+^ CD4^+^ T cells expanded in infected lungs, their transcriptional profiles revealed reduced effector gene expression, indicative of a hypo-responsive or pre-exhausted state. While our results implicate insufficient OX40L on APCs as a limiting factor, this remains a correlative observation. Definitive mechanistic confirmation will require targeted approaches, such as APC-specific restoration of OX40L expression, to directly test whether impaired ligand availability constrains OX40-mediated T cell immunity during *Mtb* infection.

OX40L is typically upregulated on APCs, such as dendritic cells and macrophages in response to acute infection or inflammation, and has a known central role in CD4^+^ T cell activation, survival, and differentiation.^24^^,^^35^ We identified, however, consistently minimal expression of OX40L in the *Mtb*-infected lung, contrasting with other chronic inflammatory conditions where elevated OX40L expression supports robust T cell responses.^36^^,^^37^^,^^38^ This persistently low expression likely reflects features of the chronically inflamed pulmonary microenvironment rather than active suppression by the pathogen, as we did not observe direct evidence of *Mtb*-mediated downregulation in our dataset. Still, *Mtb* may exploit this deficiency to limit APC-T cell interactions, thereby weakening co-stimulatory signaling essential for effective CD4^+^ T cell activation and expansion. These findings highlight a potential immune-evasion strategy and underscore the therapeutic relevance of restoring OX40 signaling to reverse T cell dysfunction.

Therapeutic administration of an OX40 agonist enhanced host resistance to *Mtb* by promoting IFN-γ-producing CD4^+^ T cells and reducing bacterial burden. These effects align with previous studies demonstrating that OX40 signaling facilitates T cell expansion and maintenance during chronic infections.^26^^,^^39^^,^^40^^,^^41^ Notably, Gress et al. reported increased lung inflammation when αOX40 treatment was initiated at week 4 and pathology assessed immediately after dosing,^26^ whereas, in our study, treatment began at day 10 and pathology was evaluated 2 weeks later, coinciding with improved bacterial control and reduced inflammation. These differences in treatment timing and evaluation likely account for the divergent histological outcomes and emphasize the stage-dependent impact of OX40 stimulation. Our single-cell analysis also did not reveal elevated Nr4a1 expression in the OX40^+^ CD4^+^ subset, despite robust OX40 expression, suggesting regulatory differences that may not be captured at the 2- to 4-week stages.

Our findings support a mechanistic model in which CD40/CD40L signaling plays a crucial role, specifically in the context of therapeutic OX40 stimulation, in regulating OX40-mediated CD4^+^ T cell responses during *Mtb* infection. CD40 is a well-established activator of dendritic cells, licensing them to provide the co-stimulatory signals required for T cell priming.^42^^,^^43^^,^^44^ In TB, a setting defined by persistent antigen exposure and immunosuppressive conditions, sustained dendritic cell (DC) activation is particularly important for maintaining protective T cell responses.^10^^,^^45^ We found that effective OX40-driven immunity requires intact CD40/CD40L signaling: blockade of CD40L abrogated OX40’s protective effects and reduced OX40 expression, indicating that CD40 signaling may stabilize OX40 expression and function, potentially via cytokine sensitization or transcriptional regulation. This is consistent with prior evidence that CD40L enhances survival and co-stimulatory receptor expression in activated T cells.^31^ Although αOX40 expanded antigen-experienced OX40^+^ CD4^+^ clones and coincided with reduced colony-forming units (CFU), protection at the individual clonotype level cannot be inferred without functional validation, such as TCR reconstruction or adoptive transfer. Furthermore, our analyses were restricted to 2–4 weeks, and whether αOX40 promotes long-lived memory remains unresolved. While some CD4^+^ clusters expressed memory-associated genes (*Tcf7*, *Lef1*, *Il7r*) and TRM-like features (*CD69*, *CXCR6*), canonical memory subsets were not clearly resolved at these early stages. Future studies at later time points with dedicated readouts will be needed, and evidence from more chronic infection models supports evaluating OX40 function at later stages of infection.^26^

Moreover, OX40 stimulation resulted in the expansion of clonally enriched, antigen-experienced CD4^+^ T cells and increased TCR repertoire diversity rather than promoting *de novo* priming from naive precursors. Such diversification could broaden epitope coverage, reduce immune escape, and enhance response durability.^46^ These effects were lost when CD40-CD40L signaling was blocked, underscoring its central role in shaping both the magnitude and quality of the T cell response. Notably, similar correlations between increased TCR diversity and improved clinical outcomes have been observed in cancer immunotherapy settings,^47^^,^^48^^,^^49^ suggesting that broadening the antigen-specific T cell repertoire may be a generalizable strategy for enhancing immune control. This parallel highlights shared immunological constraints in chronic infections and tumors, such as antigen persistence, T cell exhaustion, and impaired co-stimulation, and it raises the possibility that immunotherapeutic principles established in oncology, including agonist antibodies and repertoire reshaping, could be repurposed to improve host immunity against TB.

While OX40 agonism alone can reinvigorate dysfunctional T cells, its efficacy may be limited under conditions of impaired APC function. Therefore, therapeutic strategies that combine OX40 stimulation with agents that enhance CD40 signaling may provide a more effective means of reinvigorating T cell immunity in TB. This mirrors approaches in oncology, where co-targeting OX40 and CD40 pathways synergistically restores T cell function and enhances long-term immune control.^50^^,^^51^ Future translation will require optimization of delivery route and dose, treatment timing relative to OX40 upregulation, and potential combination with standard antibiotics. Translation to human TB remains to be established. Validation in human patient samples and *ex vivo* systems, including HIV co-infected cohorts, will be essential to assess the clinical potential of targeting the CD40-OX40 axis. Moreover, our study focused on early to mid-infection in mice before fully organized granulomas formed. Whether OX40 stimulation can overcome barriers in mature granulomas, such as limited APC access and hypoxia, remains unknown and will require evaluation in advanced models.

In conclusion, our study identifies defective OX40-OX40L engagement as a key contributor to CD4^+^ T cell dysfunction during *Mtb* infection and reveals that this impairment is governed by upstream CD40-CD40L signaling. Together, these findings define a CD40-OX40-IFN-γ axis that is essential for sustaining protective T cell responses in the TB-infected lung. This mechanistic insight provides a foundation for developing host-directed immunotherapies aimed at restoring co-stimulatory function in chronic infection. More broadly, our results support the concept that selectively targeting co-stimulatory checkpoints may offer durable immune reprogramming in TB and other persistent infections.

## Materials and methods

### Animals and *Mtb* infection

Female C57BL/6 mice (6–8 weeks old) were purchased from Beijing Vital River Laboratory Animal Technology. C3HeB/FeJ (C3H) mice (6–8 weeks old) were obtained from The Jackson Laboratory and maintained under specific pathogen-free conditions at the Animal Facility of Shenzhen University. Mice were aerosol infected with ∼200 CFU of *Mtb*. In the first two infections of this study, day 1 CFU validation confirmed ∼200 bacteria per lung, and all subsequent experiments were performed using the same standardized procedure. For time-course experiments, lungs from C57BL/6 mice were collected at 2 and 4 weeks post infection. *Rag1*^−/−^ and *Ifngr1*^−/−^ mice (C57BL/6 background, purchased from Cyagen Biosciences) were used under identical housing and infection conditions. All animal procedures were approved by the Institutional Animal Care and Use Committee (IACUC) of Shenzhen University.

### *In vivo* antibody treatments

Mice were treated intraperitoneally with 100 μg per mouse of the following monoclonal antibodies: agonistic anti-OX40 (clone OX86, Bio X Cell), anti-CD40L (blocking, clone MR1, Bio X Cell), anti-OX40L (blocking, clone RM134L, Bio X Cell), or respective isotype controls. Antibodies were administered on days 10, 12, 14, and 16 post infection. For co-treatment experiments, αOX40 and αCD40L were co-administered at equimolar concentrations. For CD4^+^ T cell depletion, anti-CD4 (clone YTS 177, Bio X Cell; 200 μg per dose) was injected every other day starting 2 days before infection and continuing until the experimental endpoint.

### Single-cell RNA and TCR sequencing

For the infection kinetics study, lungs from uninfected, 2-week, and 4-week post-infection mice were collected, perfused with PBS to reduce intravascular contents, then enzymatically digested in RPMI-1640 with 1 mg/mL collagenase D and 0.1 mg/mL DNase I (Roche) at 37°C for 30 min. Digests were filtered through 70-μm strainers, and CD45^+^ cells were enriched using magnetic microbeads (Stemcell, #18945). Viable cells were counted by trypan blue exclusion and loaded onto the 10X Genomics Chromium Controller using the Single Cell 3′ v2 kit. Single-cell libraries were prepared as previously described.^52^ Libraries were prepared per manufacturer instructions and sequenced on an Illumina NovaSeq 6000. Raw data were processed with Cell Ranger v6.0.1 (10X Genomics) using the mm10 genome reference.

For treatment comparison experiments (αIgG, αOX40, αOX40 + αCD40L), lung CD45^+^ immune cells were isolated at 4 weeks post infection and processed using the Singleron GEXSCOPE Single Cell Immune Profiling Kit, enabling simultaneous transcriptome and paired TCR V(D)J library construction. Libraries were sequenced on the NovaSeq 6000 and processed using Singleron’s sctoolkit pipeline.

### scRNA-seq data processing and clustering

Downstream analysis was performed in Python using Scanpy v1.8.2. Cells with fewer than 200 detected genes or with top 2% gene counts were excluded. The data were log normalized and scaled, and the top 2,000 variable genes were identified. Dimensionality reduction was performed using principal-component analysis (PCA), and clustering was performed with the FindClusters function, using the top 20 principal components (PCs) (resolution = 1.2). Uniform manifold approximation and projection (UMAP) plots were generated for visualization. Cell clusters were annotated based on canonical markers.

### Trajectory and pathway analysis

Differentiation trajectories of CD4^+^ and CD8^+^ T cells were reconstructed using partition-based graph abstraction (PAGA) via the scanpy.tl.paga module (Scanpy v1.8.2).^53^ Cell-cell connectivity graphs were computed using Leiden clustering and projected onto UMAP embeddings. PAGA was applied to log-normalized, PCA-reduced data (top 50 PCs), with a connectivity threshold of 0.05. RNA velocity was computed in stochastic mode using scVelo (v0.2.4) and overlaid on PAGA paths. Trajectories were validated by examining marker-gene expression along pseudotime.

Differentially expressed genes (DEGs) along trajectories were analyzed for functional enrichment using clusterProfiler (v4.0.0) for GO Biological Process and KEGG pathways. Enrichments were visualized with dot plots showing gene ratio, adjusted *p* values (Benjamini-Hochberg), and gene counts. Gene set enrichment analysis (GSEA) was performed using the fgsea package on pre-ranked logFC gene lists.

### Expression pattern clustering

To identify dynamic transcriptional programs over the course of infection, time-series clustering was performed using the Mfuzz package (v2.46.0) in R.^54^ Samples were grouped by disease stage (uninfected, 2 weeks, 4 weeks). Genes with >25% missing values were excluded; for the remaining genes, missing values were imputed using the gene’s mean expression. Data were preprocessed using filter.std and standardize functions. Fuzzy c-means clustering partitioned genes into nine clusters based on temporal trajectories.

### Ligand-receptor interaction analysis

CellPhoneDB v4.0.0 and the LIANA framework (https://github.com/saeyslab/liana) were used to infer cell-cell communication from normalized scRNA-seq data. Interactions with *p* < 0.05 and expression in >10% of cells in each cluster were retained. Results were visualized as dot plots showing mean expression and adjusted *p* values for selected ligand-receptor pairs, with a focus on co-stimulatory and inhibitory axes.

### scTCR-seq analysis

Clonotypes were assigned based on identical paired CDR3 nucleotide sequences of TCR α and β chains. Clonal expansion was defined as clonotypes comprising more than two cells and accounting for ≥0.2% of all TCR clonotypes within each sample. TCR diversity was quantified using the immunarch package (Chao1 richness, D50 index). TCR-specificity clusters were identified using GLIPH2 (https://github.com/immunoengineer/gliph2) with filtered CDR3β input sequences (>8 amino acids, vbScore <0.05). Circos plots were generated to show clonal overlap between cell clusters.

### Flow cytometry

Lung single-cell suspensions were stained with fluorophore-conjugated antibodies (listed in Table S8). Surface markers included CD45, CD3, CD4, CD8, CD11b, Ly6G, Siglec-F, OX40, OX40L, and CD40. For cytokine detection, intracellular IFN-γ staining was performed using the BD Cytofix/Cytoperm Plus Fixation/Permeabilization Solution Kit with BD GolgiStop (catalog no. 554715). Data were acquired on a BD FACSAria II flow cytometer and analyzed with FlowJo v10.8.

### T cell-macrophage co-culture and CFU assay

BMDMs were generated by culturing bone marrow cells in DMEM with 10% FBS and 20 ng/mL macrophage colony stimulating factor (M-CSF) (PeproTech) for 7 days. BMDMs were infected with *Mtb* H37Rv (MOI = 5) for 4 h, washed, and co-cultured with FACS-sorted lung CD3^+^CD4^+^OX40^+^ T cells at a 4:1 ratio. Cultures were treated with αOX40 (10 μg/mL) or αIgG (10 μg/mL) and incubated for 72 h. Bacterial loads were assessed by lysing macrophages with 0.1% Triton X-100 and plating on 7H10 agar supplemented with 10% oleic acid-albumin-dextrose-catalase (OADC). Colonies were counted after 3–4 weeks of incubation at 37°C.

### Bacterial load quantification

For *in vivo* CFU assays, lung and spleen homogenates were serially diluted in PBS with 0.05% Tween-80 and plated in triplicate on Middlebrook 7H10 agar with OADC. Plates were incubated at 37°C for 3–4 weeks, and colonies were manually counted.

### Histology

Lungs were inflated with 10% neutral-buffered formalin, fixed overnight, paraffin embedded, sectioned at 5 μm, and stained with hematoxylin and eosin (H&E). Images were acquired using a Hamamatsu NanoZoomer whole-slide scanner.

### Statistical analysis

Statistical analyses were performed using GraphPad Prism v9.0 unless otherwise indicated. Data are presented as mean ± SEM. Two-group comparisons were analyzed using unpaired *t* tests, while comparisons involving three or more groups were analyzed using one-way ANOVA. A *p* value <0.05 was considered statistically significant.

## Data availability

All data associated with this study are included in the paper or the supplemental information. The sequencing raw data and processed data used in this paper are available in the Genome Sequence Archive (GSA) at the Beijing Institute of Genomics (BIG) Data Center, Chinese Academy of Sciences (BIG: CRA026252). Any additional information required to reanalyze the data reported in this work is available from the corresponding author upon request.

## Acknowledgments

We are grateful to the Biosafety Level 3 Laboratory of Shenzhen University for providing essential support to this study. This work was supported by grants from the Shenzhen Medical Research Funding Program (nos. B2302035 and A2302004), the 10.13039/501100001809National Natural Science Foundation of China (nos. 82430074 and 82472294), the National Key Research and Development Program of China (no. 2022YFC2302901), the Science and Technology Innovation Commission of Shenzhen (no. JSGG20220822095200001), and the Shenzhen University 2035 Program for Excellent Research (no. 2023B005).

## Author contributions

Y.C. and X.Z. conceived the study. X.Z., J.Y., F.L., Yu Wang, C.G.F., X.C., Y.C., F.P., and W.W. developed the methodology. Y.C., Q.Y., X.Z., J.Y., F.L., and Y.H. performed the investigations. Z.L., Yejun Wang, Y.C., and C.S. contributed to data visualization. X.C. and Y.C. acquired funding, administered the project, and supervised the work. X.C., Y.C., X.Z., and F.P. wrote the original draft, and all authors contributed to reviewing and editing the manuscript.

## Declaration of interests

The authors declare no competing interests.

## Supplemental information

Document S1. Figures S1–S7

Table S1. Marker genes of all subsets identified by scRNA-seq during *Mtb* infection

Table S2. Co-expressed gene clusters in T cell identified by time-series clustering during *Mtb* infection

Table S3. T cell subsets classification by unsupervised clustering during *Mtb* infection

Table S4. scRNA-seq-based classification of immune cell clusters across co-stimulatory treatments

Table S5. T cell subsets annotation across co-stimulatory treatments

Table S6. Summary of paired TCR sequences identified in treated *Mtb*-infected mice

Table S7. Clonotype grouping identified by GLIPH2 in treated *Mtb*-infected lung T cells

Table S8. Antibodies used for flow cytometric analysis of lung immune cells

Document S2. Article plus supplemental information
