# Supplementary material for: A CD40-OX40 co-stimulatory circuit orchestrates protective CD4+ T cell immunity in tuberculosis
Source: Mol Ther. 2026 Jan 3;34(4):2468–81. doi: 10.1016/j.ymthe.2025.12.062 (PMC13069330; doi:10.1016/j.ymthe.2025.12.062)
Supplement: Supplementary file 1 [file mmc1.pdf]

YMTHE, Volume *34*

Supplemental Information

A CD40-OX40 co-stimulatory circuit orchestrates protective $CD4^+$ T cell immunity in tuberculosis

Ximeng Zhang, Jing Yang, Fuxiang Li, Yu Wang, Zhaodong Li, Wenfei Wang, Yunlong Hu, Chenyan Shi, Fan Pan, Carl G Feng, Yejun Wang, Qianting Yang, Xinchun Chen, and Yi Cai

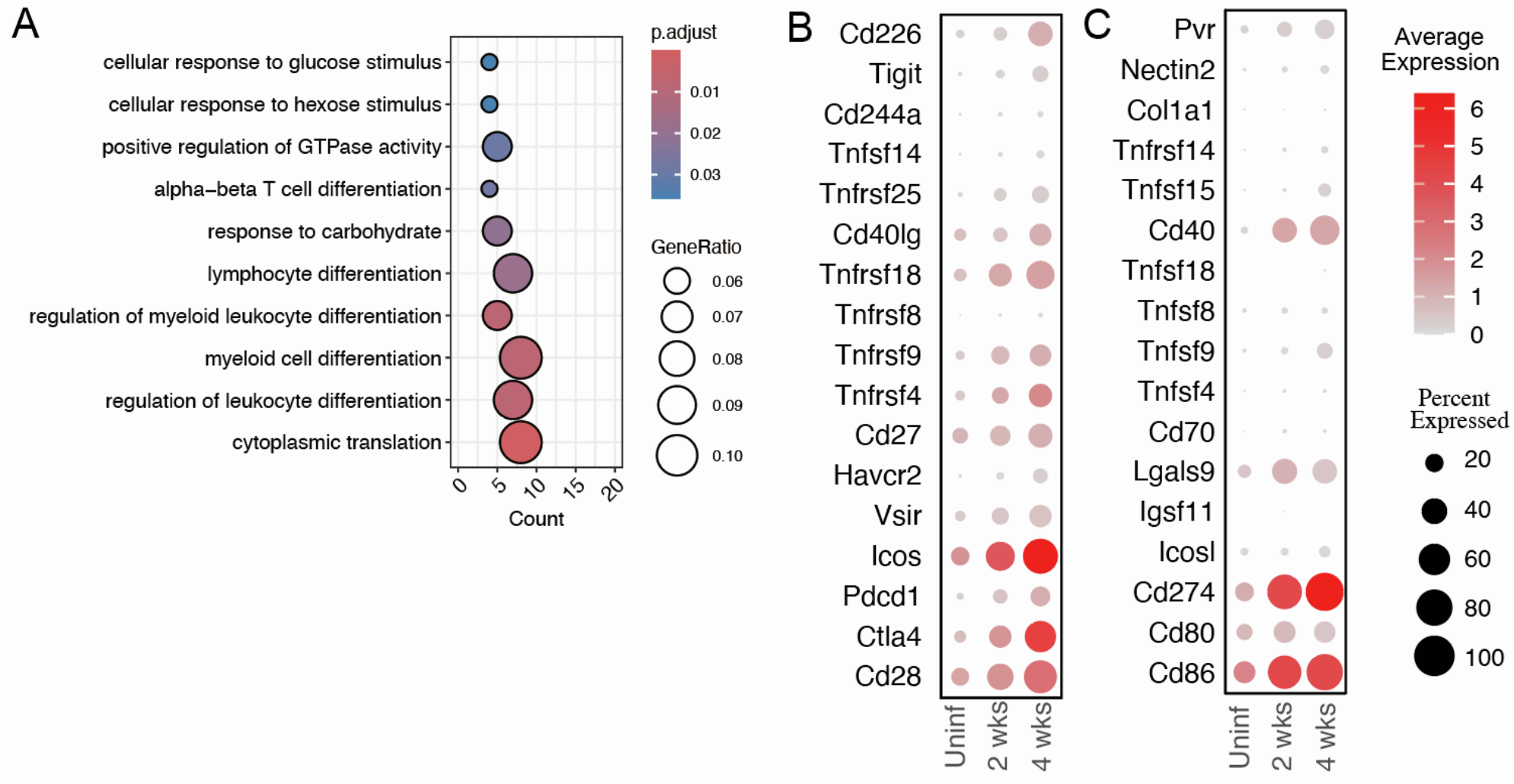

Figure S1. Functional enrichment and co-signaling molecule expression in lung T and myeloid cells during Mtb infection. (A) GO enrichment analysis of genes from Cluster 7 during Mtb infection. Dot size indicates gene ratio; color scale reflects adjusted *p*-values. (B) Dot plot of co-stimulatory and co-inhibitory receptor genes in T cells at Uninf, 2-week, and 4-week time points. Dot size corresponds to the percentage of cells expressing each gene; color indicates average expression level. (C) Expression profile of corresponding ligand genes in myeloid cells across the same time points, with dot size and color representing expression frequency and intensity as in (B).

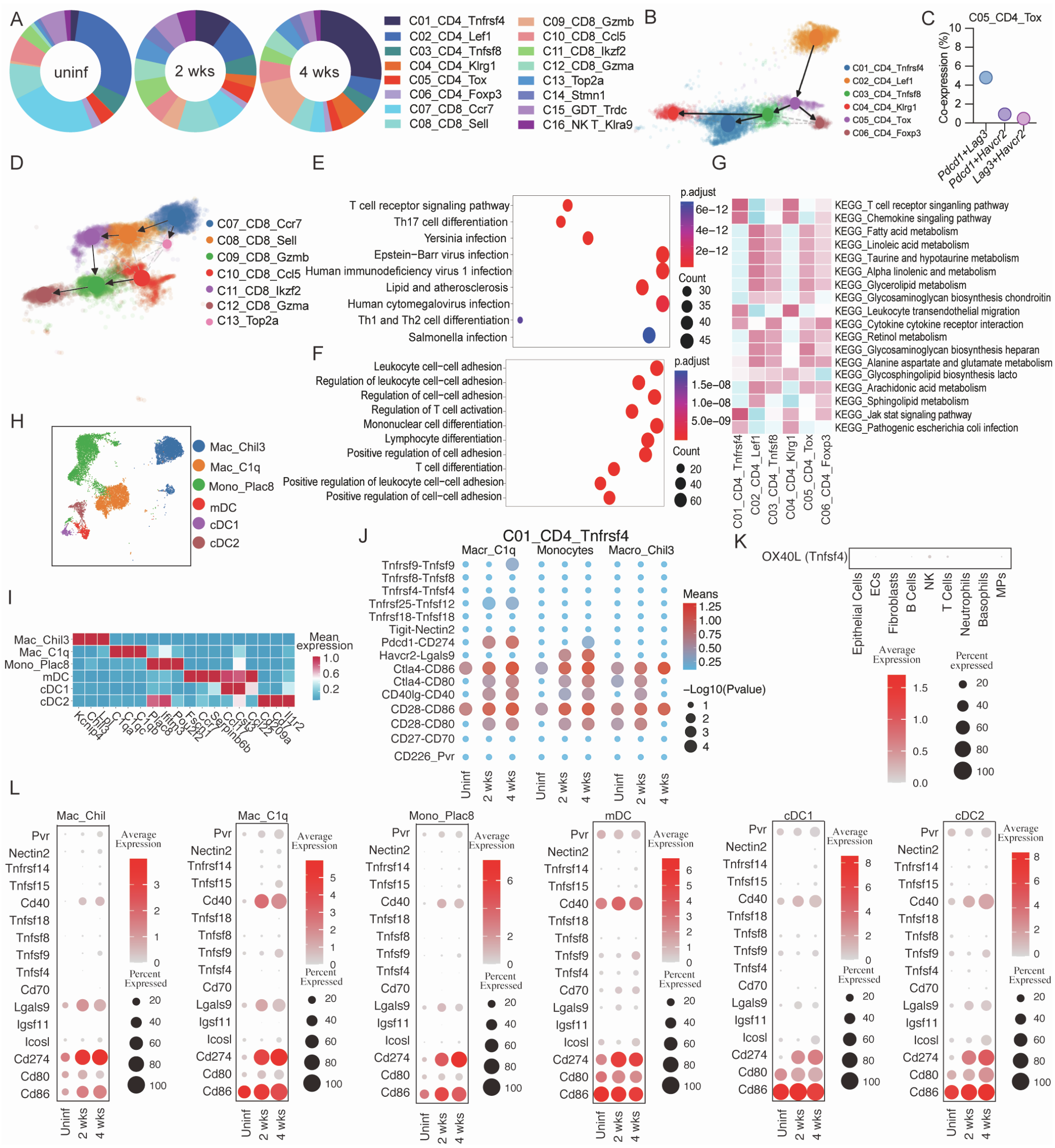

Figure S2. Single-cell profiling of lung T cells and myeloid subsets with inferred ligand–receptor interactions during *Mtb* infection. (A) Donut plots of annotated T cell clusters across uninfected, 2-week, and 4-week groups. (B) Trajectory analysis of lung CD4 T cell subsets using PAGA (partition-based graph abstraction), visualized on a UMAP embedding and colored by cluster identity. (C) Co-expression frequencies of inhibitory receptors *Pdcd1* (PD-1), *Lag3*, and *Havcr2* (TIM-3) within the C05_CD4_TOX cluster. (D) Trajectory analysis of lung CD8 T cell subsets using PAGA, visualized on a UMAP embedding and colored by cluster identity. (E–F) GO enrichment analyses upregulated (E) and downregulated (F) genes in the C01_CD4_Tnfrsf4 subset compared with other CD4^{+} T cell subsets. (G) Heatmap of pathway activity

scores for KEGG terms across CD4$^+$ T cell subsets. (H) UMAP plot of annotated myeloid populations, including macrophage subsets (Mac_Chi3, Mac_C1q), monocytes (Mono_Plac8), and dendritic cells (mDC, cDC1, cDC2). (I) Heatmap of selected gene expression profiles across annotated myeloid cell clusters. (J) Dot plot of predicted ligand–receptor interactions between myeloid subsets and the C01_CD4_Tnfrsf4 T cell cluster at different time points. (K) Dot plot of Tnfsf4 (encoding OX40L) expression across major immune and non-immune cell populations in the lung. (L) Dot plots of co-stimulatory and co-inhibitory ligand gene expression across individual myeloid subpopulations (Mac_Chi3, Mac_C1q, Mono_Plac8, mDC, cDC1, cDC2) in uninfected, 2-week, and 4-week infected lungs.

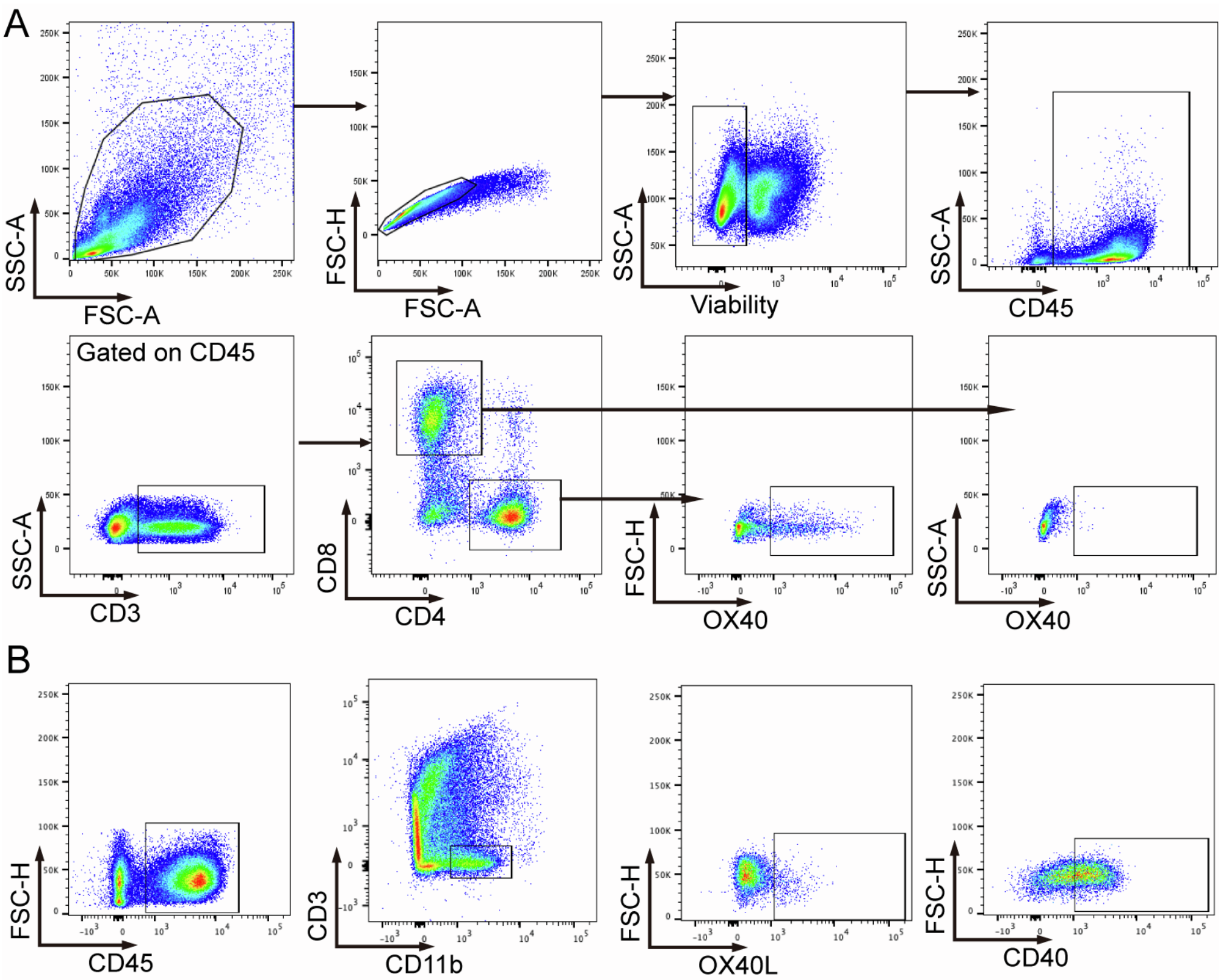

Figure S3. Flow cytometry gating strategy for identifying OX40 and co-signaling molecule expression in *Mtb*-infected mouse lung immune cells. (A) Sequential gating strategy for identifying OX40 expression on CD4^{+} and CD8^{+} T cells from lung single-cell suspensions. (B) Gating strategy for detecting OX40L and CD40 expression on myeloid cell populations.

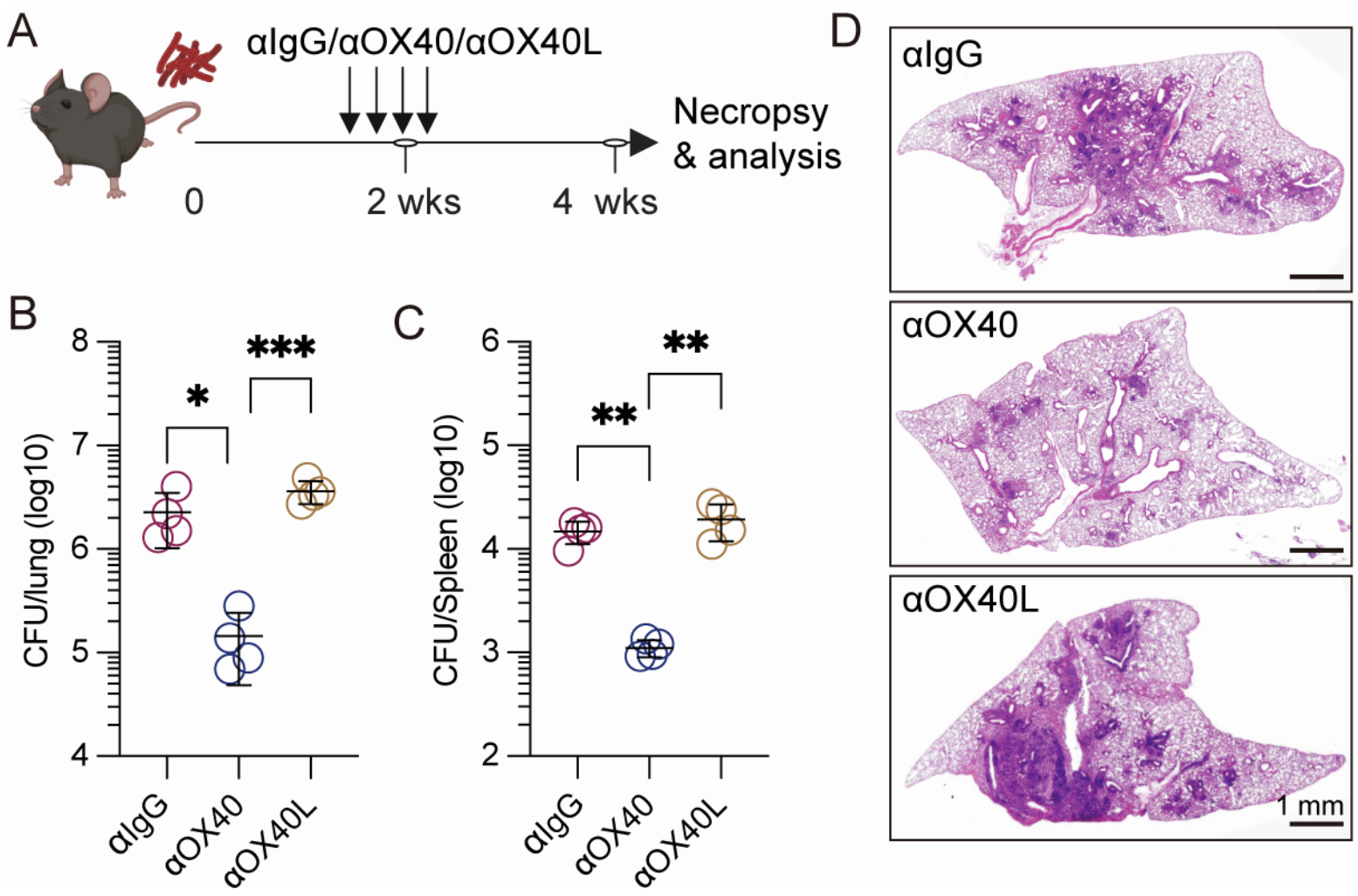

Figure S4. Comparison of αOX40 and αOX40L treatment during *Mtb* infection in mice. (A) Schematic overview of the treatment groups (αIgG, αOX40, αOX40L) and timing of sample collection at 4 weeks. (B–C) Bacterial burden in the lungs (B) and spleens (C) measured as CFU per organ across treatment groups (n = 4). (D) Representative H&E-stained lung sections from each group at 4 weeks. Data are presented as mean ± SEM. Statistical comparisons were performed using one-way ANOVA. **p*<0.05, ***p*<0.01, ****p*<0.001.

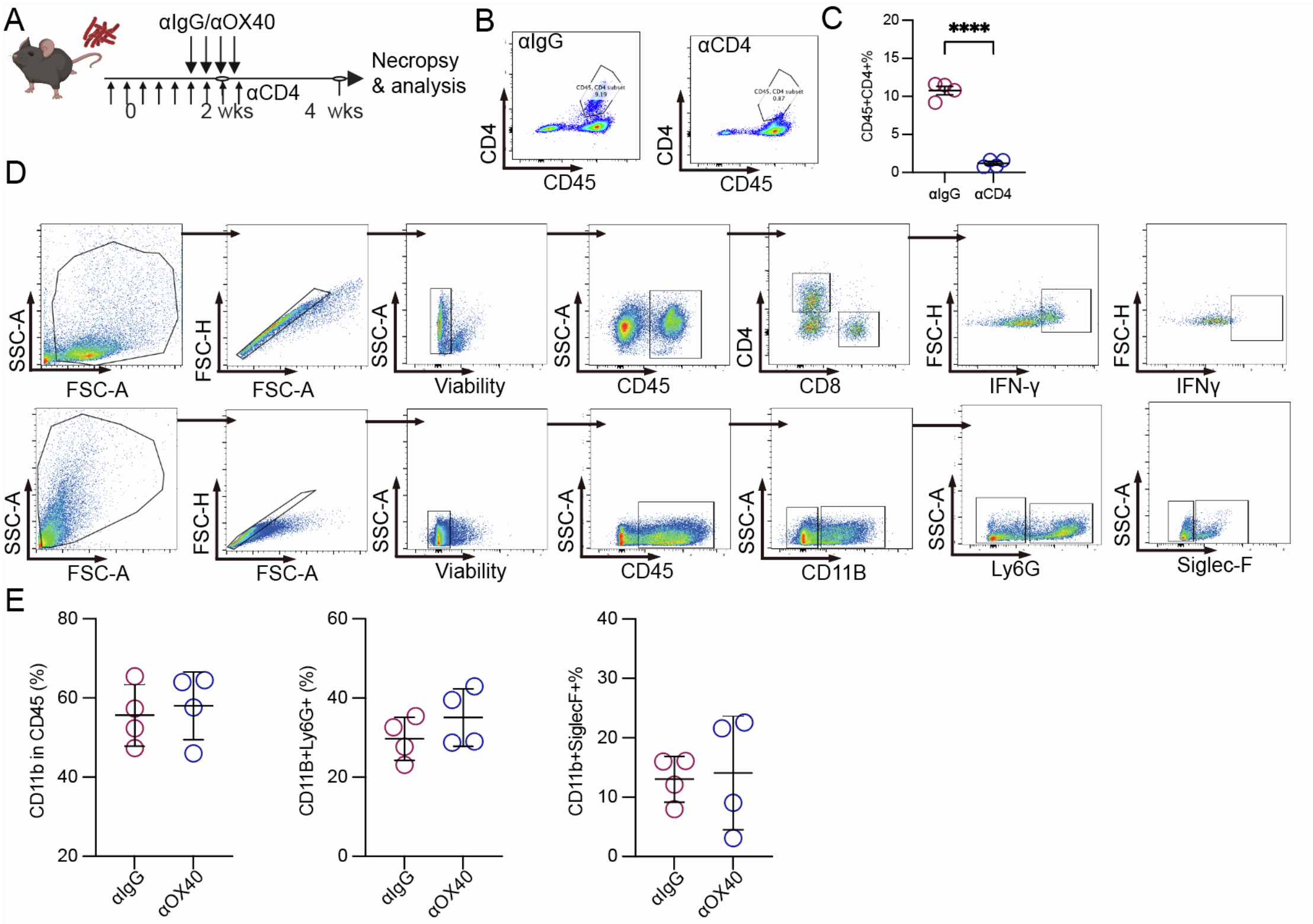

Figure S5. Flow cytometric validation of CD4⁺ T-cell depletion and analysis of immune populations following αOX40 treatment. (A) Diagram of the treatment groups (αIgG, αOX40, αCD4) and the experimental timeline. (B–C) Representative flow cytometry plots (B) and quantification (C) of $CD4^+$ T-cell depletion (n = 4). (D) Gating strategy for identifying IFN-γ^+ T cells (top) and myeloid subsets (bottom) in lung samples. (E) Quantification of total $CD11b^+$ cells among $CD45^+$ populations, $Ly6G^+$ neutrophils among $CD11b^+$ cells, and Siglec-F^+ eosinophils within the $CD11b^+$ compartment in αIgG- and αOX40-treated mice (n = 4).

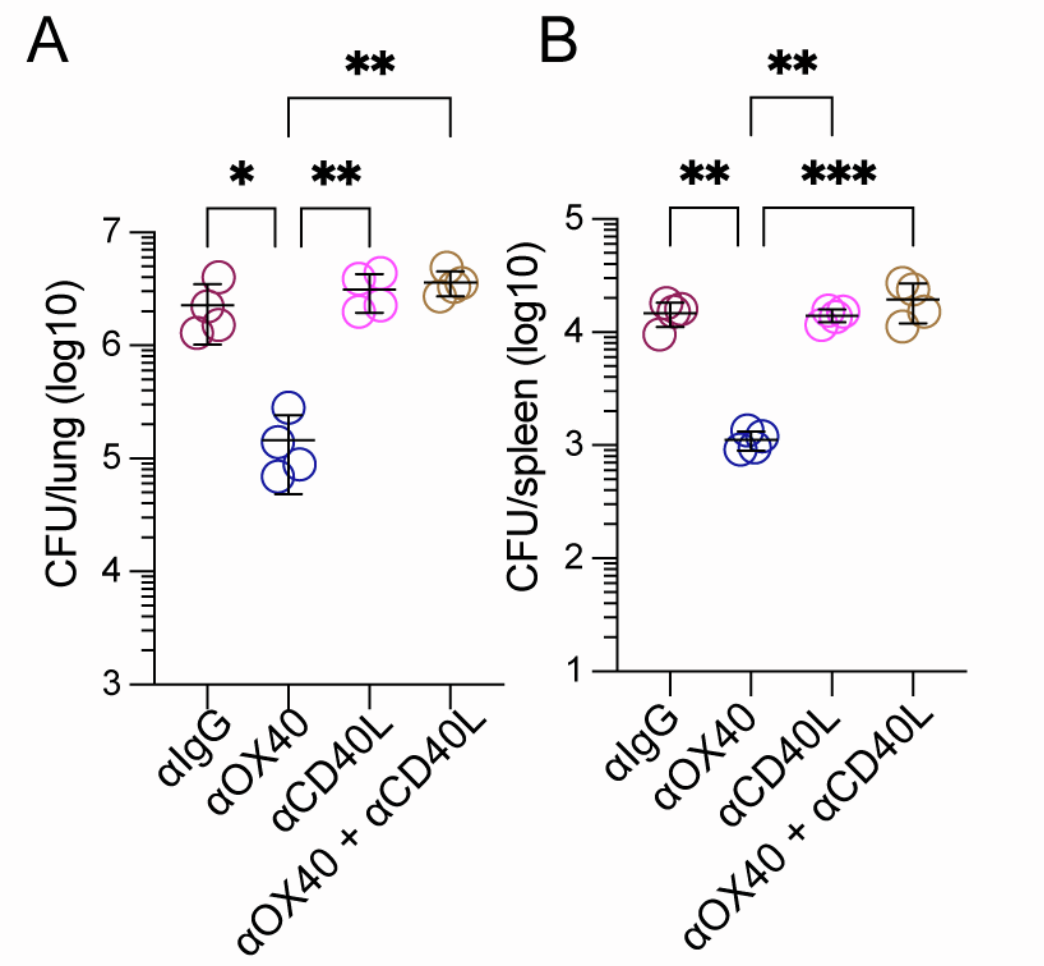

Figure S6. Bacterial burden in lungs and spleens of *Mtb*-infected mice treated with single or combined αOX40 and αCD40L. (A–B) CFU quantification in the lungs (A) and spleens (B) of Mtb-infected mice treated with αIgG, αOX40, αCD40L, or a combination of αOX40 + αCD40L, assessed at 4 weeks post-infection (n = 4). Data are presented as mean ± SEM. Statistical analysis was performed using one-way ANOVA. **p*<0.05, ***p*<0.01, ****p*<0.001.

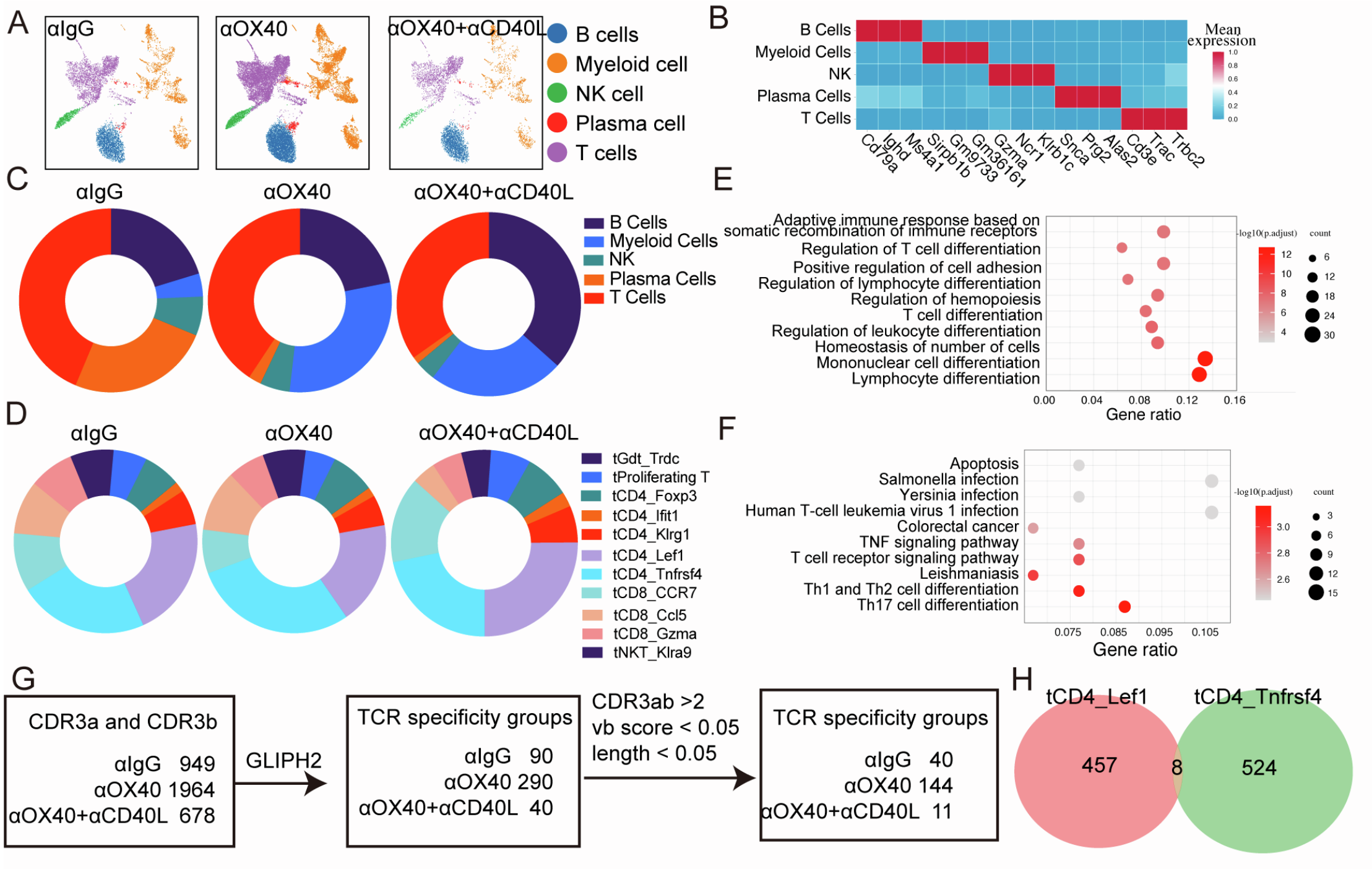

Figure S7. Single-cell transcriptomic and TCR profiling of lung immune cells following αOX40 and αCD40L treatment. (A) UMAP projection of major immune cell populations from lungs of mice treated with αIgG, αOX40, or αOX40 + αCD40L, colored by annotated cell type. (B) Heatmap of lineage-defining genes used to annotate immune cell types. (C) Donut plots of B cells, myeloid cells, NK cells, plasma cells, and T cells in each treatment group. (D) Donut plots of T cell clusters across treatment conditions. (E) GO enrichment analysis of genes upregulated in the tCD4_Tnfrsf4 subset from αOX40- versus αIgG–treated mice. (F) KEGG pathway enrichment analysis of genes upregulated in the tCD4_Tnfrsf4 subset from αOX40- versus IgG-treated mice. (G) Summary of GLIPH2 analysis of CDR3α and CDR3β sequences recovered and the resulting TCR specificity groups identified under each treatment condition. (H) Venn diagram comparing TCR clonotypes between the tCD4_Lef1 and tCD4_Tnfrsf4 clusters.

Supplemental tables

Table S1 Marker genes of all subsets identified by scRNA-seq during Mtb infection

Table S2 Co-expressed gene clusters in T cell identified by time-series clustering during Mtb infection

Table S3 T cell subsets classification by unsupervised clustering during Mtb infection

Table S4 scRNA-seq-based classification of immune cell clusters across co-stimulatory treatments

Table S5 T cell subsets annotation across co-stimulatory treatments

Table S6 Summary of paired TCR sequences identified in treated Mtb-infected mice

Table S7 Clonotype grouping identified by GLIPH2 in treated Mtb-infected lung T cells

Table S8 Antibodies used for flow cytometric analysis of lung immune cells
